# Immigrant Tortricidae: Holarctic versus Introduced Species in North America

**DOI:** 10.3390/insects11090594

**Published:** 2020-09-03

**Authors:** Todd M. Gilligan, John W. Brown, Joaquín Baixeras

**Affiliations:** 1 USDA-APHIS-PPQ-S&T, 2301 Research Boulevard, Suite 108, Fort Collins, CO 80526, USA; 2 Department of Entomology, National Museum of Natural History, Smithsonian Institution, Washington, DC 20560, USA; tortricidae.jwb@gmail.com; 3 Institut Cavanilles de Biodiversitat i Biologia Evolutiva, Universitat de València, Carrer Catedràtic José Beltran, 2, 46980 Paterna, Spain; joaquin.baixeras@uv.es

**Keywords:** Olethreutinae, Tortricinae, DNA barcoding, Beringian, taxonomy

## Abstract

**Simple Summary:**

The family Tortricidae includes approximately 11,500 species of small moths, many of which are economically important pests worldwide. A large number of tortricid species have been inadvertently introduced into North America from Eurasia, and many have the potential to inflict considerable negative economic and ecological impacts. Because native species behave differently than introduced species, it is critical to distinguish between the two. Unfortunately, this can be a difficult task. In the past, many tortricids discovered in North America were assumed to be the same as their Eurasian counterparts, i.e., Holarctic. Using DNA sequence data, morphological characters, food plants, and historical records, we analyzed the origin of 151 species of Tortricidae present in North America. The results indicate that the number of Holarctic species has been overestimated by at least 20%. We also determined that the number of introduced tortricids in North America is unexpectedly high compared other families, with tortricids accounting for approximately 23–30% of the total number of moth and butterfly species introduced to North America. This suggests that introduced tortricids have a greater potential of becoming economically important pests than moths in other families, and why distinguishing Holarctic from introduced species is critical to American agriculture.

**Abstract:**

In support of a comprehensive update to the checklist of the moths of North America, we attempt to determine the status of 151 species of Tortricidae present in North America that may be Holarctic, introduced, or sibling species of their European counterparts. Discovering the natural distributions of these taxa is often difficult, if not impossible, but several criteria can be applied to determine if a species that is present in both Europe and North America is natively Holarctic, introduced, or represented by different but closely related species on each continent. We use DNA barcodes (when available), morphology, host plants, and historical records (literature and museum specimens) to make these assessments and propose several taxonomic changes, as well as future areas of research. The following taxa are raised from synonymy to species status: *Acleris ferrumixtana* (Benander, 1934), stat. rev.; *Acleris viburnana* (Clemens, 1860), stat. rev.; *Acleris pulverosana* (Walker, 1863), stat. rev.; *Acleris placidana* (Robinson, 1869), stat. rev.; *Lobesia spiraeae* (McDunnough, 1938), stat. rev.; and *Epiblema arctica* Miller, 1985, stat. rev. *Cydia saltitans* (Westwood, 1858), stat. rev., is determined to be the valid name for the “jumping bean moth,” and *Phiaris glaciana* (Möschler, 1860), comb. n., is placed in a new genus. We determine that the number of Holarctic species has been overestimated by at least 20% in the past, and that the overall number of introduced species in North America is unexpectedly high, with Tortricidae accounting for approximately 23–30% of the total number of Lepidoptera species introduced to North America.

## 1. Introduction

Globalization, the international movement of commodities and people among different nations, has dramatically increased the spread of plant and animal species around the world, e.g., [1,2,3]. Among these “introduced” or “exotic” species, insects are the most pervasive, representing 87% of the approximately 2500 nonnative terrestrial invertebrates in Europe [4]. According to Lovett et al. [5], nonnative insect species have accumulated in United States forests at a rate of approximately 2.5 per year over the last 150 years, with the gypsy moth (*Lymantria dispar* (Linnaeus, 1758), Erebidae) being among the most notorious. Examples of important lepidopteran pests that have been introduced recently to new regions around the globe include the fall armyworm, *Spodoptera frugiperda* (J. E. Smith) (Noctuidae), a native of the New World that has spread to much of Africa and Asia [6,7] and was recently discovered in Australia [8]; the Old World bollworm, *Helicoverpa armigera* (Hübner) (Noctuidae), which was first reported in the New World in Brazil and has spread to much of South America and the Caribbean [9,10,11]; the light brown apple moth, *Epiphyas postvittana* (Walker) (Tortricidae), a native of Australia that was documented from California in 2006 and has now spread throughout much of the state [12]; and the European grapevine moth, *Lobesia botrana* (Denis and Schiffermüller) ([Tortricidae]), a native of Europe that was inadvertently introduced to the wine-growing regions of Argentina, Chile, and California [13]. While these contemporary or recent arrivals to new regions are well documented, for many other species, there is considerable ambiguity regarding their origin and/or native distributions. For example, in North America, the discovery of a “European” species could indicate that the taxon was recently introduced, that it is natively Holarctic in distribution and previously undiscovered, or that it represents an unrecognized sibling species of its European counterpart. In the absence of direct evidence of an introduction, the taxonomist is left to speculate among these alternative explanations. In many cases, a compelling explanation is further complicated by the fact that many apparently European species are discovered along the northwestern or northeastern coasts of North America, regions where one would expect to find Holarctic elements or introductions owing to the proximity to major U.S. and Canadian ports of entry.

In support of a comprehensive update to the checklist of the moths of North America [14], we determine the status of 151 species of Tortricidae present in North America that have been previously assumed to be Holarctic, or introduced, or whose status in the Nearctic was questionable. We use DNA barcodes (when available), morphology, host plants, and historical records (literature and museum specimens) to make these assessments, and we propose appropriate taxonomic changes where necessary.

## 2. Materials and Methods

### 2.1. Biogeographic Framework

**The Holarctic**. The Holarctic is defined as the biogeographical region comprised of the Nearctic (North America) and the Palearctic (Eurasia and northern Africa). These continents have been variably linked since the breakup of Pangea approximately 180 mya. The following geographic history of the region is summarized from Hopkins [15], Enghoff [16], and Sanmartín et al. [17]. Following the breakup of Pangea, North America and Eurasia comprised the northern supercontinent Laurasia, which was split into two smaller paleocontinents, Euramerica (Europe and eastern North America) and Asiamerica (Asia and western North America) around 100–80 mya. The western Palearctic was separated from the eastern Palearctic by the Turgai Sea until ca. 30 mya, and the western Nearctic was separated from the eastern Nearctic until ca. 60 mya by the Mid-Continental Seaway. Europe and eastern North America were linked by several North Atlantic land bridges until sometime in the late Eocene (ca. 39 mya). Asia and western North America were connected by the Bering land bridge from the mid-Cretaceous (100 mya) continuously until the Late Pliocene (3.5 mya), but this connection was established again intermittently throughout the Pleistocene (1.5–0.011 mya) before it was submerged completely ca. 11,000 years ago.

These Trans-Beringian land bridges are assumed to have played an important role in dispersal across the Holarctic. Beringia underwent three distinct phases with associated changes in climate and vegetation [17]. Beringian Bridge I: A continuous belt of boreotropical forest extended over the entire Northern Hemisphere by the Early Eocene (ca. 50 mya), and the climate was much warmer and more humid than today. Beringian Bridge II: As the climate cooled and became drier, the forests transitioned to mixed deciduous hardwoods and conifers, and eventually only to conifer forests that were split into eastern and western portions. The marine transgression of the Bering Strait in the Late Pliocene (3.5 mya) permanently separated the Palearctic and Nearctic forests. Beringian Bridge III: As glaciation commenced in the Pleistocene (1.5–1.0 mya), the Beringian land bridge was once again established. Originally assumed to be a continuous “mammoth steppe” or “steppe tundra,” Elias et al. [18] found that the vegetation was dominated by birch-heath-graminoid tundra with little or no evidence of steppe elements. This land connection persisted intermittently during glaciation events until it was permanently interrupted at the end of the Pleistocene.

It is generally assumed that trans-Beringian dispersal between the eastern Palearctic and western Nearctic is more frequent than trans-Atlantic dispersal events. Sanmartín et al. [17] found that in the Early Tertiary (70–45 mya), the North Atlantic connection was more important than the Beringian connection, but that faunal exchange was more frequent across the second Beringian Bridge (20–3.5 mya), and much more frequent across the third Beringian Bridge (1.5–0.011 mya). This is similar to what has been proposed for plants, where Beringia was more important than the North Atlantic bridges since the Middle Eocene (45 mya) [19]. During full-glacial periods in the Pleistocene, Beringia was essentially part of Siberia, with the unglaciated parts of Alaska and the Yukon separated from the rest of North America, which was largely covered in ice sheets. As glaciers receded across North America, Palearctic or Beringian insects had the opportunity to disperse from former refugia, events that have occurred relatively recently. When examining ant species, Schär et al. [20] determined that all three of the naturally occurring Holarctic ant species had dispersed from the Palearctic no more than 2 mya. Studies of Noctuidae have found that most naturally occurring (e.g., not introduced) Holarctic species are associated with the tundra zone, and all truly Holarctic noctuid species are present somewhere in Beringia [21].

**Holarctic or Introduced?** Determination of whether a species currently found in both Eurasia and North America is Holarctic versus introduced may, in many cases, represent an unsolvable puzzle. Although the association of a species with Beringia lends support to a Holarctic distribution, other factors require consideration in determining a species’ natural distribution. These factors include the organism’s current range, its host plant use, available historical records, morphological and/or molecular variation, etc.

Factors that would be expected for a naturally occurring Holarctic species include the following: (1) no direct evidence of introduction; (2) association with native occurring host plants; (3) first reported in noncoastal, inland areas; (4) no evidence of range expansion in recent times; and (5) presence in the Arctic biogeographical region.

Factors associated with introductions, as would be expected for taxa that were transported by man between or among continents, include the following: (1) direct evidence of introduction; (2) association with nonnative or frequently imported host plants; (3) first documented in a coastal area; (4) evidence of range expansion in recent times; and (5) absence from the Arctic biogeographical region.

Although the above criteria can be applied to hypothesize a taxon’s natural distribution, the most important factor may lie in an initial determination of whether more than one species is involved. Early taxonomists in North America often sought the opinions of their European counterparts. For example, in Tortricidae, the opinions of Lord Walsingham and P. C. Zeller, European authorities in the latter half of the nineteenth and early twentieth centuries, strongly influenced the taxonomy of New World species and genera. Superficial similarity between a Nearctic and Palearctic species was sometimes enough to assign them to the same taxon, particularly before the discovery of the value of genitalia for identification. One of the most striking examples of a North American taxon hiding under a European name is the Nearctic *Paralobesia viteana* (Clemens) and the Palearctic *Lobesia botrana*. Both moths are pests of grape and have similar wing patterns, but their genitalia are very different, and a recent phylogenetic study has shown that *Lobesia* and *Paralobesia* are indeed separate genera [22]. In a paper on the North American species *viteana*, Zeller [23] determined that it was the same as the European *botrana* and synonymized them, with *botrana* as the senior name. What was far from understood at the time was that the early concept of *P. viteana* included numerous other species of *Paralobesia*, all with similar wing patterns. A recent revision of this genus resulted in the identification of 40 different species in North America [22]; thus Zeller had inadvertently synonymized up to 40 different Nearctic taxa under a single European name, none of which were actually Holarctic. The advent of studying genitalia helped to resolve some of the more obvious problems with taxon assignment in Tortricidae; however, genitalia were not always a panacea, as the amount of morphological variation in these structures was not well studied, a problem that remains today. Thus, even Heinrich [24,25], in his two monographs on North American Olethreutinae in which he examined genitalia for nearly every species, incorrectly identified some endemic Nearctic taxa as similar European species.

### 2.2. Taxa Evaluated

The list of tortricid taxa examined for this study was obtained primarily from the Checklist of the Moths of North America [14] and the World Catalogue of Tortricidae [26], with updates from the web-based catalogue [27], the preliminary “P3” updated checklist of the Lepidoptera of North America [28], and unpublished notes and partial manuscripts prepared by the late William E. Miller. Numerous other publications were referenced for each individual taxon; these are listed in the species accounts. Determination of the “previous status” for each species was obtained primarily from the checklist of Pohl et al. [28], with some minor modifications based on our knowledge of particular taxa or obvious mistakes in the “P3” list. Taxon order follows the “P3” list and the updated North American checklist that is in preparation. The single institutional abbreviation used in the text is USNM = U.S. National Museum of Natural History, Smithsonian Institution, Washington, DC, USA.

### 2.3. Molecular Methods

Although the use of genitalia in lepidopteran systematics was a giant step forward, the use of molecular data represents an even greater advancement. While new DNA sequencing technologies continue to make the sequencing entire genomes more affordable, even simple DNA barcodes can be highly informative. DNA barcodes [29] represent an excellent source of molecular data, complementary to morphology, because sequences are easy to obtain, even from older museum specimens, and a large database of reference sequences has been assembled over the last decade (Barcode of Life Database; BOLD). Patterns in DNA sequences can be used as evidence regarding the status of morphologically similar populations in Europe and North America, either directly or through the discovery of morphological characters that corroborate similarities in DNA sequences. Within Tortricidae, several recent studies have used DNA barcoding to determine if two or more entities represent a single Holarctic taxon or are similar species occurring on different continents, e.g., [30,31,32,33].

As with genital morphology, DNA barcodes are not without shortcomings with regards to their ability to identify species boundaries. Early studies (e.g., [29,34]) proposed fixed distance thresholds between species (e.g., 2%) and “barcoding gaps” between different taxa. Although these early concepts have been largely discarded (see summary in [35]), DNA barcodes remain an extremely informative source of evidence of genetic similarity among closely related species.

DNA barcode sequences were obtained from BOLD. All records for a particular taxon, usually at the genus level, were selected with the option “include Public” records. Records were filtered to exclude contaminants, records with stop codons, and records flagged as misidentifications or errors. Only sequences ≥400 bp were used because of random alignment issues with shorter sequences. Sequences were aligned in BOLD using the BOLD amino acid-based HMM Aligner, and neighbor-joining trees were constructed in BOLD using the Kimura 2 Parameter (K2P) distance model.

The “revised status” of each taxon was determined by examining how the specimens clustered in the distance tree, the identity and origin of the specimens, the specimens themselves (photos in BOLD or actual specimens), the relevant literature, and additional statistics in BOLD. In many cases, the average and maximum p-distance for each of BOLD’s Barcode Index Numbers (BINs) and the p-distance between the nearest neighbors were examined; these are reported in the results when relevant. We did not use a specific percentage cut-off for species delimitation. We also did not rely exclusively on BOLD’s BIN assignments, although in most cases, the placement of more than one taxon in a single BIN or the splitting of one taxon into multiple BINs did indicate there was an issue, either with the taxonomy or identifications. In some cases, the DNA data was completely ambiguous, either due to a lack of data, too many potential misidentifications, or the apparent sharing of DNA barcodes across multiple taxa.

## 3. Results and Discussion

Each of the following species accounts includes five components: (1) the previously concluded status of the species with regards to its biogeographic origin (“Previous status”), usually as listed in Pohl et al. [28]; (2) our determination of its status based on the criteria we applied (“Revised status”); (3) whether our assessment is supported by DNA barcodes currently in the BOLD database (“Supported by DNA in BOLD”); (4) the BIN (Barcode Index Number from BOLD) into which putative conspecific specimens are clustered (absent for taxa where there are no sequence data); and (5) a brief text discussion in which we present the evidence upon which our determination is based.

**Family TORTRICIDAE**

**Subfamily TORTRICINAE**

***Acleris forsskaleana* (Linnaeus, 1758)**

**Previous status:** Introduced from Europe

**Revised status:** No change

**Supported by DNA in BOLD:** Yes

**BINs:** BOLD:AAA8796

Sequences of *A. forsskaleana* group into a single BIN (BOLD:AAA8796) with representatives from North America (Ontario, British Columbia, Pennsylvania) and Europe (Norway, the United Kingdom, Austria, Germany, Czech Republic, Italy, Hungary, Finland). Many of the specimens from both continents share identical barcodes. *Acleris forsskaleana* was first recorded from North America by Klots [36], who examined specimens collected from Long Island, New York in 1934. Powell and Burns [37] discovered that the earliest specimens from that location were actually collected in 1932, and they provide a detailed account of this species spreading in the northeastern United States.

***Acleris holmiana* (Linnaeus, 1758)**

**Previous status:** Introduced from Eurasia

**Revised status:** No change

**Supported by DNA in BOLD:** Yes

**BINs**: BOLD:AAE9876

Sequences of *A. holmiana* form a single BIN, with specimens from Europe (Germany, Norway, Finland, Italy) and North America (Washington, British Columbia). There is little sequence variation, and many specimens share identical barcodes. The first records of *A. holmiana* in North America are from the Lower Fraser Valley, British Columbia in 1977 [38,39]. Miller (unpublished) estimated the actual introduction to be three years earlier (1974).

***Acleris comariana* (Zeller, 1846)**

**Previous status:** Introduced from Eurasia

**Revised status:** No change; potential cryptic species in eastern North America

**Supported by DNA in BOLD:** Yes

**BINs:** BOLD:AAA6998 (Europe, Canada; *A. comariana*); BOLD:AAA6997 (eastern North America; *Acleris* sp.)

Sequences of specimens identified as *A. comariana* fall into two BINs: BOLD:AAA6998 consists of specimens from Canada (British Columbia) and Europe (Finland, Norway, United Kingdom), while BOLD:AAA6997 consists of specimens from eastern North America (Quebec, Ontario, Tennessee, Illinois, Virginia). Both BINs form distinct clusters and are separated by >7.7% (p-dist). The first BIN represents *A. comariana*, the second BIN likely represents a cryptic species in eastern North America that has been consistently misidentified as *A. comariana*.

*Acleris comariana* was first introduced to British Columbia, with the earliest record from Richmond in 1972 [40,41]. Belton [42] states that it was introduced much earlier, i.e., in 1924 from Belgium, but we could not find evidence to substantiate this claim. This species is recorded from Japan as early as 1917 [43], so it could have been introduced to North America from Europe or Asia.

***Acleris rhombana* ([Denis and Schiffermüller], 1775)**

**Previous status:** Holarctic?

**Revised status:** Introduced from Eurasia

**Supported by DNA in BOLD:** Yes

**BINs:** BOLD:AAE3741

Sequences of *A. rhombana* are placed in a single BIN (BOLD:AAE3741) representing specimens from Canada (British Columbia) and Europe (Austria, Finland, Italy, The Netherlands, Norway, United Kingdom). Many of the specimens from both continents share identical barcodes. Razowski [44] lists the natural distribution of *A. rhombana* as Europe and Asia Minor. The first report of this species occurring in North America is from Obraztsov [45], although he provides no other details. Razowski [44] cites this paper and suggests that *A. rhombana* was artificially introduced to North America. It is possible that this species is Holarctic and was simply not discovered in North America prior to the mid-1950s. However, Pohl et al. [46] record this species from British Columbia and Nova Scotia, with the majority of specimens collected near Victoria on Vancouver Island. Both southwestern British Columbia and Nova Scotia are areas where introduced taxa are frequently encountered, and there appear to be no records prior to Obraztsov’s [45] report. As such, we believe this taxon is likely introduced to North America.

***Acleris notana* (Donovan, [1806])**

**Previous status:** Introduced from Europe

**Revised status:** Introduced?

**Supported by DNA in BOLD:** No (no specimens from North America in BOLD)

**BINs:** BOLD:AAB4490

Specimens of *A. notana* in BOLD are placed in a single BIN (BOLD:AAB4490) with representatives from Europe (Austria, Finland, Germany, Norway, United Kingdom). These form a distinct cluster close to sequences of *A. ferrugana* from Europe (under which *A. notana* was previously synonymized). Razowski [47] states that *A. notana* was introduced into North America. Pohl et al. [46] state that records of *A. notana* from North America may refer to *A. arcticana* (see [48]), and that Winn [49] used the name *A. ferrugana* for records of *A. notana* from Canada. The DNA data are not sufficient to support or refute any of these statements because there are no specimens from North America of this taxon in BOLD.

***Acleris implexana* (Walker, 1863)**

**Previous status:** Holarctic

**Revised status:***Acleris implexana* (Nearctic); *Acleris ferrumixtana* (Benander, 1934), **stat. rev.** (Holarctic)

**Supported by DNA in BOLD:** Yes

**BINs:** BOLD:AAB6464 (Canada; *A. implexana*); BOLD:AAB6465 (Europe, Canada; *A. ferrumixtana*)

Sequences in BOLD identified as *A. implexana* are placed into two BINs: BOLD:AAB6464 contains specimens from Canada (Alberta, British Columbia, Manitoba, New Brunswick, Quebec, Ontario, Saskatchewan), and BOLD:AAB6465 contains specimens from Canada (Manitoba, Ontario, Quebec, Yukon Territory) as well as Europe (Finland, Norway). The two BINs appear to represent distinct species, as they are separated by 3.4% (p-dist) and do not form a single cluster.

*Acleris implexana* was described from northern Ontario. *Acalla ferrumixtana* was described from Scandinavia and placed in *Acleris* by Obraztsov [45], where it remained a separate species until it was treated as a subspecies of *A. implexana* by Razowski (e.g., [47,50]). Razowski [47] lists two subspecies of *A. implexana*: the nominotypical one from the Nearctic and *A. implexana ferrumixtana* from the Palearctic. The two subspecies can be separated based on the shape of the sterigma. From the sequence data, it appears that *A. implexana* is Nearctic and represented by BOLD:AAB6464, and *A. implexana ferrumixtana* is a separate species represented by BOLD:AAB6465 that is Holarctic in distribution. Based on the evidence provided here, we resurrect *A. ferrumixtana* (Benander, 1934), stat. rev., to species status and assign the specimens represented by BOLD:AAB6465 to this name.

***Acleris schalleriana* (Linnaeus, 1761)**

**Previous status:** Holarctic

**Revised status:***Acleris schalleriana* (Palearctic); *Acleris viburnana* (Clemens, 1860), **stat. rev.** (Nearctic)

**Supported by DNA in BOLD:** Yes

**BINs:** BOLD:AAG9608 (Europe); BOLD:AAB2825 (North America)

Sequences identified as *A. schalleriana* cluster into two BINs: BOLD:AAG9608 contains specimens from Europe (Germany, Norway, Bulgaria, and Finland) and BOLD:AAB2825 contains specimens from Canada and the United States. The two BINs are separated by 2.9% (p-dist) and together form a single cluster.

Razowski [47] treats *A. schalleriana* as Holarctic with a different subspecies in Europe and North America (i.e., nominotypic *schalleriana* in Europe, *viburnana* in North America). Based on the sequence data, it is likely that the Palearctic and Nearctic populations represent different species. As such, we resurrect *A. viburnana* (Clemens, 1860), stat. rev., to species status and assign the specimens represented by BOLD:AAB2825 to this name. *Acleris schalleriana* is represented by BOLD:AAG9608 and restricted to the Palearctic.

***Acleris variegana* ([Denis and Schiffermüller], 1775)**

**Previous status:** Introduced from Eurasia

**Revised status:** No change

**Supported by DNA in BOLD:** Yes

**BINs**: BOLD:AAB2294 (North America, Europe); BOLD:ACE3007 (Europe)

Sequences of *A. variegana* are placed in two BINs: BOLD:AAB2294 contains specimens from Canada, the United States, and Europe (Austria, Italy, The Netherlands, Norway, United Kingdom), while BOLD:ACE3007 contains specimens from Europe (Czech Republic, Finland, France, Germany, Italy, Norway). Both BINs form a single cluster and are separated by only 0.97% (p-dist). As such, we assume these different BINs represent population-level structuring and not distinct taxa. Specimens from North America and Europe share identical barcodes. *Acleris variegana* was introduced from Eurasia to North America, with the earliest record of this species from a specimen collected in Victoria, British Columbia in 1920 [41].

***Acleris hastiana* (Linnaeus, 1758)**

**Previous status:** Holarctic

**Revised status:***Acleris hastiana* (Palearctic); *Acleris pulverosana* (Walker, 1863), **stat. rev.** (Nearctic)

**Supported by DNA in BOLD:** Yes

**BINs**: BOLD:AAA9796 (Europe; *A. hastiana*); BOLD:AAA9793 (Canada; *A. pulverosana*); BOLD:AAA9792 (Canada; *A. pulverosana*); BOLD:AAA9794 (Canada; unknown, not *A. hastiana*)

Sequences of *A. hastiana* are distributed across four BINs: BOLD:AAA9796 contains specimens from Europe (Austria, Bulgaria, Croatia, Finland, Germany, Italy, Norway, Sweden, United Kingdom), while BOLD:AAA9793 contains specimens from the Pacific Northwest collected near Seattle and Vancouver. BOLD:AAA9792 contains specimens from Canada (Alberta, British Columbia, New Brunswick, Ontario, Yukon Territory). BOLD:AAA9794 contains six specimens from Canada (Alberta, Manitoba).

The first three BINs containing sequences labeled as *A. hastiana* group into two main clusters, with specimens from Europe (BOLD:AAA9796) forming one cluster, and North America (BOLD:AAA9793 and BOLD:AAA9792) forming the second. However, all three BINs are separated by at least 3.3% (p-dist). These three BINs are well separated from BOLD:AAA9794 by > 4.5% (p-dist). This could indicate that there are potentially three distinct taxa in North America, or that specimens in some of the BINs are consistently misidentified (but see below). Regardless, it appears that the European taxon is separate from those in North America.

Razowski [47] states that *A. hastiana* is Holarctic and distributed in North America from Canada to California. The DNA data suggest that *A. hastiana* is restricted to the Palearctic, and that populations in the Nearctic represent at least one additional species. Razowski [47] also lists several “closely related” species in the Holarctic: *A. youngana* (McDunnough, 1934); *A. robinsoniana* (Kearfott, 1904); and *A. britannia* (Kearfott, 1904). There are examples of each of the latter three species in BOLD that are placed into BINs separate from any of the *A. hastiana* sequences. Thus, it does not appear that North American *A. hastiana* sequences were simply misidentified as another species of *Acleris*. There is a synonym of *A. hastiana* described from Canada: *A. pulverosana* (Walker, 1863). Based on the DNA evidence, we raise *A. pulverosana* (Walker, 1863), stat. rev., from synonymy and assign the North American populations formerly identified as *A. hastiana* to this name. Further research will be necessary to determine the status of specimens in BOLD:AAA9794.

***Acleris arcticana* (Guenée, 1845)**

**Previous status:** Holarctic

**Revised status:** No change

**Supported by DNA in BOLD:** Yes

**BINs:** BOLD:ACF4284 (Canada, Europe, Greenland); BOLD:ACF5694 (Canada); BOLD:AAD0128 (Canada)

The BOLD database contains three BINs with specimens identified as *A. arcticana*: BOLD:ACF4284 represents specimens from Greenland, Yukon Territory, and Norway; BOLD:ACF5694 represents specimens from Canada (Manitoba, Ontario, Quebec); and BOLD:AAD0128 represents specimens from Ontario. Specimens in the latter two BINs are identified as “*Acleris* sp. 1;” however, all three BINs form a single cluster that appears to represent a single species.

***Acleris robinsoniana* (Forbes, 1923)**

**Previous status:** Holarctic?

**Revised status:** Nearctic only

**Supported by DNA in BOLD:** Yes

**BINs:** BOLD:AAD6538

Sequences of *A. robinsoniana* cluster in a single BIN (BOLD:AAD6538) that includes specimens from Canada and the U.S. Although this species is extremely variable and can appear similar to many other species of *Acleris* [51], there are no records from outside of North America. *Acleris robinsoniana* is widely distributed in the eastern U.S., the Pacific Northwest, and much of Canada.

***Acleris logiana* (Clerck, 1759)**

**Previous status:** Holarctic

**Revised status:***Acleris logiana* (Palearctic); *Acleris placidana* (Robinson, 1869), **stat. rev.** (Nearctic)

**Supported by DNA in BOLD:** Yes

**BINs:** BOLD:AAB0755 (*A. logiana*); BOLD:AAB0754 (*A. placidana*)

Two BINs contain sequences from specimens currently identified as *A. logiana*: BOLD:AAB0755 contains specimens form Europe (Austria, Finland, Germany, Norway), and BOLD:AAB0754 contains specimens from the U.S. and Canada. These two BINs are separated by >2.49% (p-dist) and segregated by geographic location, i.e., one from Europe and one from North America. Razowski [47] stated that Nearctic populations of *A. logiana* are treated as the subspecies *placidana*. The DNA data suggests that these should be considered distinct species, so we elevate *A. placidana* (Robinson, 1869), stat. rev., to species status. The current world catalogue [27] inadvertently made this change several years ago; however, this was simply a mistake in translating the subspecies status of *placidana* from the original publication [26]. Pohl et al. [28] followed this treatment in their North American checklist. We formalize the treatment of *A. placidana* as a separate taxon here to avoid confusion. *Acleris trisignana* Robinson, 1869, described from West Virginia, remains a synonym of *A. placidana*.

***Acleris maccana* (Treitschke, 1835)**

**Previous status:** Holarctic

**Revised status:** No change

**Supported by DNA in BOLD:** Yes

**BINs:** BOLD:AAA8391

Sequences in BOLD identified as *A. maccana* are contained in a single BIN (BOLD:AAA8391) with specimens from North America (Canada and the United States) and Europe (Austria, Czech Republic, Finland, Norway). These sequences form a distinct cluster, supporting the prior assumptions that *A. maccana* is Holarctic [44,45,51].

***Acleris lipsiana* ([Denis and Schiffermüller], 1775)**

**Previous status:** Holarctic? (from historical records)

**Revised status:***Acleris lipsiana* (Palearctic); *Acleris capizziana* Obraztsov, 1963 (Nearctic)

**Supported by DNA in BOLD:** Yes

**BINs:** BOLD:AAD6818 (A. lipsiana, A. capizziana)

Sequences of *A. lipsiana* fall into a single BIN (BOLD:AAD6818) However, specimens from Europe (Austria, Finland, Norway) and North America (Alberta, Quebec, Oregon, Washington) are separated into two clusters. Overall variation within the entire BIN is approximately 1.2% (p-dist). One of the specimens in the North American cluster is a paratype of *A. capizziana* Obraztsov, 1963 from Oregon.

Pohl et al. [46] indicated that North American records of *A. lipsiana* are actually *A. inana*, a taxon that Clarke [52] suggested might be synonymous with *A. flavivittana* (Clemens, 1864). DNA data suggest that the North American records of *A. lipsiana* in BOLD are *A. capizziana*. Although most specimens of *A. capizziana* appear different from those of *A. lipsiana*, the single *A. capizziana* from Alberta looks similar to *A. lipsiana* from Europe. Razowski [44] states that “*A. capizziana* Obr. is very difficult to determine when similarly coloured as *A. lipsiana* (Schiff. and Den.),” and also noted that the male genitalia of these two species are very similar.

***Acleris scabrana* ([Denis and Schiffermüller], 1775)**

**Previous status:** Holarctic

**Revised status:** No change

**Supported by DNA in BOLD:** No (no specimens from North America in BOLD)

**BINs:** BOLD:AAS0630

Only two sequences of *A. scabrana* are listed in BOLD, one from Latvia and the other from an unlisted location. Until more sequence data are generated from Eurasia and North America, there is no DNA evidence to determine the status of this species. It is currently treated as Holarctic [47], and McDunnough [53] first reported it for North America.

***Acleris effractana* (Hübner, 1822)/*Acleris emargana* (Fabricius, 1775)**

**Previous status:***Acleris effractana* (Holarctic); *Acleris emargana* (Palearctic)

**Revised status:** No change; possible cryptic species in Europe

**Supported by DNA in BOLD:** Yes

**BINs:** BOLD:AAB3524 (Europe; *A. emargana*); BOLD:AAB3525 (North America, Europe; *A. effractana*); BOLD:AAA6999 (Europe)

Sequences from specimens identified as either *A. effractana* or *A. emargana* fall into three BINs: BOLD:AAB3524 contains specimens from Europe (Austria, Germany, Finland, France, Italy, Norway); BOLD:AAB3525 contains specimens from North America (British Columbia, Quebec, Washington, Yukon Territory) and Europe (Finland, Norway); and BOLD:AAA6999 contains specimens from Finland and Norway.

Karsholt et al. [54] resolved the taxonomy of these two species. They determined that *A. emargana* and *E. effractana* could be reliably separated by morphological characters (length of the socii in the males and length of the ductus bursae in the females). *Acleris emargana* is Palearctic and distributed throughout Europe and Asia, and *A. effractana* is Holarctic and distributed throughout North Europe, northern Central Europe, Russia, Japan, Canada, and the northwestern United States [47].

DNA data support the findings of Karsholt et al. [54]. BOLD:AAB3524 represents *E. emargana* from Europe. BOLD:AAB3525 represents *E. effractana* and forms two groups within a single cluster, one containing specimens from Europe and the other containing specimens from North America. There is, however, a third BIN that likely represents a different species. BOLD:AAA6999 forms a separate cluster that is sufficiently distinct (2.75% and 3.98% p-dist) from the other two BINs to indicate it is a potential cryptic species from Finland and Norway. Specimens in BOLD included in this BIN are identified as either *A. emargana*, *A. effractana*, or *Acleris* sp.

***Cnephasia longana* (Haworth, [1811])**

**Previous status:** Introduced from Europe

**Revised status:** No change

**Supported by DNA in BOLD:** No (misidentifications and/or DNA barcodes do not separate species)

**BINs:** BOLD:AAC7742

Sequences of *C. longana* are represented in a single BIN, BOLD:AAC7742. These include specimens from North America (California, Washington) and Europe (Austria, Denmark, Germany, Norway, United Kingdom). Many of the specimens from Europe are identified as *C. genitalana* (and a few as *C. longana*). These two species can be separated by genitalia, and a few of the European specimens are dissected, indicating that at least some of the identifications in BOLD are correct. Thus, it appears that barcodes of *C. longana* and *C. genitalana* are nearly identical and cannot be used to reliably separate these two taxa.

*Cnephasia longana* was first reported from California by Pritchard et al. [55] and from British Columbia by Cram and Tonks [56]. Powell [57] indicated that it was introduced into western North America and was established in British Columbia, the Puyallup Valley in Washington, the Willamette Valley in Oregon, and around the San Francisco and Monterey Bay areas of California. The earliest record is from Oregon in 1929 on strawberries [41,57].

***Cnephasia asseclana* ([Denis and Schiffermüller], 1775)**

**Previous status:** Introduced from Europe

**Revised status:** No change

**Supported by DNA in BOLD:** Yes

**BINs:** BOLD:AAA6293

Sequences of *C. asseclana* fall into a single BIN with very little genetic diversity. BOLD:AAA6293 contains specimens from Canada (Ontario, Nova Scotia) and Europe (Austria, Finland, Germany, Italy, Macedonia, The Netherlands, Norway, United Kingdom). Mutuura [58] provided a history of *C. asseclana* (as *C. interjectana*) and *C. stephensiana* in North America. *Cnephasia asseclana* was first collected in North America in Newfoundland in 1915. These specimens were described as *Tortrix oleraceana* Gibson, 1916, and this name was later synonymized by Freeman [59] under *Cnephasia virgaureana* (Treitschke, 1835), currently a junior synonym of the Palearctic *C. asseclana*.

***Cnephasia stephensiana* (Doubleday, 1849)**

**Previous status:** Introduced from Europe

**Revised status:** No change

**Supported by DNA in BOLD:** Yes

**BINs:** BOLD:AAA6292

*Cnephasia stephensiana* is represented by a single BIN (BOLD:AAA6292), with specimens from Canada (British Columbia, New Brunswick, Ontario, Quebec) and Europe (Austria, France, Finland, Germany, Italy, Macedonia, Norway). Mutuura [58] provided a history of *C. stephensiana* and *C. asseclana* (as *C. interjectana*) in North America. This species was first discovered in North America when it was collected in Nova Scotia in 1954, and it was found in several locations in eastern Canada in the late 1970s [58]. *Cnephasia stephensiana* is currently distributed throughout the northeastern U.S. and eastern Canada.

***Eana argentana* (Clerck, 1759)**

**Previous status:** Holarctic

**Revised status:***Eana argentana* (Palearctic); North American cryptic species (Nearctic)

**Supported by DNA in BOLD:** Yes

**BINs:** BOLD:AAA8425 (Europe, North America); BOLD:ACE7529 (New Mexico)

A single BIN, BOLD:AAA8425, contains representatives of *E. argentana* from Europe and North America. Numerous other specimens identified as *E. argentana* in the *E. osseana* group (below) are apparent misidentifications. Almost none of the specimens represented in BOLD are dissected.

BOLD:AAA8425 is divided into two distinct clusters: the first includes specimens from Canada (Alberta, British Columbia, Yukon Territory) and Washington; the second includes specimens from Europe (Austria, Finland, Germany, Italy, Macedonia). The mean variation within each of these clusters is 0.14% (p-dist), but they are separated by approximately 1% (p-dist) and treated as separate entities in BOLD’s cluster analysis. The other BIN, BOLD:ACE7529, is >3.3% (p-dist) from the main BIN and almost certainly contains misidentified specimens or an undescribed species. Based on the DNA data, it appears that *E. argentana* is restricted to the Palearctic, and specimens identified as this taxon in North America represent one or more cryptic species that have yet to be described. This is not surprising, given that many of these specimens are found at higher elevations and in isolated mountain habitats.

***Eana osseana* (Scopoli, 1763)**

**Previous status:** Holarctic

**Revised status:***Eana osseana* (Palearctic); North American cryptic sp. 1 (Nearctic); North America cryptic sp. 2 (Nearctic); Holarctic cryptic sp. 3 (Holarctic)

**Supported by DNA in BOLD:** Yes

**BINs:** BOLD:AAA6265 (Europe; *E. osseana*); BOLD:AAA6264 (North America; cryptic sp. 1); BOLD:ABZ7263 (Canada; cryptic sp. 2); BOLD:AAA4129 (North America, Russia; cryptic sp. 3)

There are approximately 200 sequences representing specimens identified as *E. osseana* in BOLD. These specimens are placed in four BINs: BOLD:AAA6265 contains specimens from Europe (Austria, Finland, Germany, Italy, Norway, Slovenia, United Kingdom); BOLD:AAA6264 contains specimens from Canada (Alberta, British Columbia, Newfoundland, Nova Scotia) and the United States (Alaska and California); BOLD:ABZ7263 contains specimens from Alberta and Yukon Territory; and BOLD:AAA4129 contains specimens from Canada (British Columbia, Newfoundland, Manitoba, Alberta, and Yukon Territory), the United States (Alaska and Utah), and Russia.

The BIN from Europe, BOLD:AAA6265, forms a distinct cluster that is >3.30% (p-dist) divergent from the other *E. osseana* BINs. Its nearest neighbor is actually *E. argentana*. Based on the geographic separation of the other BINs, it is likely BOLD:AAA6265 represents “true” *E. osseana*, which was described from Slovenia. This would indicate that *E. osseana* is Palearctic in distribution and not Holarctic.

The remaining three BINs (BOLD:AAA6264; BOLD:ABZ7263; BOLD:AAA4129) form a large cluster that also contains BOLD:ACE7529 (identified as *E. argentana*) and several other *Eana* specimens that are not placed in BINs. It is likely that this cluster represents several species. The largest group, BOLD:AAA4129, represents a taxon that is possibly Beringian, given that it is distributed across Northwest Canada and Alaska to Russia (the sequences from Russia are high quality and full length, and thus appear to be placed correctly). BOLD:AAA6264 and BOLD:ABZ7263 seem to be restricted to North America and also possibly represent new taxa.

There are more than 20 junior synonyms placed under *E. osseana* currently. Thus, it is possible that at least some of these names could be applied to these other BINs. Pohl et al. [46] state that the subspecies *niveosana* “applies to all North American populations, and may warrant full status.” It is likely that this name could be applied to one of the North American groups, but without further research, it is not clear to which BIN it should be assigned. Similar to *E. argentana*, many of these specimens are found at higher elevations and genitalic variation is high in this group. Further research will be necessary to resolve these *Eana* species complexes.

***Aethes deutschiana* (Zetterstedt, 1840)**

**Previous status:** Holarctic

**Revised status:***Aethes deutschiana* (Palearctic); several potential cryptic species in North America

**Supported by DNA in BOLD:** Yes

**BINs:** BOLD:AAB4420 (France, Norway, Finland; *A. deutschiana*); BOLD:ACJ1175 (Alberta); BOLD:ACE5554 (Alberta, British Columbia); BOLD:AAB4419 (Manitoba); BOLD:ACE5515 (Washington, Alberta, British Columbia); BOLD:ACE5553 (Yukon Territory, British Columbia)

Specimens of *A. deutschiana* are placed in several BINs: five BINs contain specimens from North America and a single BIN (BOLD:AAB4420) contains specimens from Europe. The five BINS form two distinct clusters, with one representing Europe and the other North America. Thus, it appears that *A. deutschiana* is Palearctic and not present in North America. The large amount of genetic diversity in the North American cluster suggests the possibility of several cryptic species. There are two junior synonyms of *A. deutschiana* described from Labrador that could potentially be applied to one or more of the North American BINs.

***Aethes rutilana* (Hübner, 1818)**

**Previous status:** Holarctic?

**Revised status:** Holarctic

**Supported by DNA in BOLD:** Yes

**BINs:** BOLD:AAC1488

Sequences of *A. rutilana* form a single BIN (BOLD:AAC1488), with shallow splits between North American representatives (i.e., British Columbia and Quebec) and two European clusters that overlap in geographic distribution. Razowski [60] described the subspecies *Aethes rutilana canadiana* from specimens from Quebec, Ontario, Manitoba, and Alberta, and though he did not explicitly state so, it seems as though he intended the name to represent a North American subspecies. *Aethes rutilana* was introduced into British Columbia sometime prior to 1965 [41].

***Aethes smeathmanniana* (Fabricius, 1781)**

**Previous status:** Holarctic

**Revised status:** No change

**Supported by DNA in BOLD:** Yes

**BINs:** BOLD:AAB1945

All sequences of *A. smeathmanniana* reside in a single BIN (BOLD:AAB1945), with specimens from Europe and North America, indicating it is likely Holarctic, as concluded by Razowski [60]. In the Nearctic region, this species is recorded from Canada (Alberta, British Columbia, Quebec, Yukon Territory) and the U.S. (California, Maine, Minnesota, Montana, Ohio, New Jersey). In the Palearctic region, it ranges across the northern latitudes (e.g., Finland, Norway, Sweden) and higher elevations (e.g., the alps of Italy and Switzerland).

***Agapeta zoegana* (Linnaeus, 1767)**

**Previous status:** Introduced from Europe

**Revised status:** No change

**Supported by DNA in BOLD:** No (genetic variability of this species is unknown)

**BINs:** BOLD:AAA6574 (United Kingdom); BOLD:AAA6573 (Finland, Norway); BOLD:ABA1841 (Macedonia); BOLD:AAA6572 (Canada); BOLD:ACJ6816 (Italy); BOLD:AAA6575 (Austria, Germany)

Sequences of *A. zoegana* are placed in six BINs in BOLD. There is a significant amount of variation between BINs (averaging 3% p-dist), but all six BINs form a single cluster. It is not known if this variation is indicative of different geographic populations of the same species, or if one or more cryptic species are involved. Regardless, the taxon currently recognized as *A. zoegana* has a well-documented history of being released in North America as a biocontrol agent.

According to Story [61] and Story et al. [62], specimens of *A. zoegana* from Austria and Hungary were introduced near Stevensville, Montana in July 1984 for the biological control of star-thistles (*Centaurea* sp.; Asteraceae), which had become a weedy pest in rangelands. By 1991, *A. zoegana* was well established in Montana, and according to Wilson and Randall [63], it is now present “in most western states.” We have verified records from Montana, Washington, and British Columbia.

***Cochylidia subroseana* (Haworth, [1811]**)

**Previous status:** Holarctic

**Revised status:** No change

**Supported by DNA in BOLD:** No (no specimens from North America in BOLD)

**BINs:** BOLD:AAD2823 (Europe)

Sequences of *C. subroseana* form a single BIN (BOLD:AAD2823) that includes specimens from Italy, Austria, Finland, Norway, and South Korea; there are no sequence data from North American specimens. Although Pogue [64] considered our single North American representative to be an undescribed species, Razowski [60] reported it from Ontario and Alberta as *Cochylidia subroseana*. We examined specimens from Colorado that appear to be conspecific with *C. subroseana*. Hence, we suspect that it is indeed Holarctic. In the Palearctic, this species is widely distributed from the United Kingdom and Norway across northern and central Europe to China.

***Cochylis atricapitana* (Stephens, 1852)**

**Previous status:** Introduced from Europe

**Revised status:** No change

**Supported by DNA in BOLD:** No (no specimens from North America in BOLD)

**BINs:** BOLD:ABZ6437 (Russia); BOLD:ABZ6441 (Russia); BOLD:AAE1271 (Norway, Denmark); BOLD:ABZ6440 (United Kingdom, The Netherlands)

Sequences of *C. atricapitana* are represented by four BINs in BOLD, which include specimens from Europe (Denmark, The Netherlands, Norway, Russia, United Kingdom). There are no sequence data for any North American specimens. Each of the BINs contains only a few specimens, and they all form a single cluster; we do not know if this indicates a single variable species or multiple taxa. According to De Clerck-Floate and Cárcamo [65], this species was introduced into British Columbia from Europe in 1990 for biocontrol of tansy ragwort (*Jacobaea vulgaris*; Asteraceae).

***Neocochylis*** ***dubitana* (Hübner, 1799)**

**Previous status:** Holarctic

**Revised status:** No change

**Supported by DNA in BOLD:** Yes

**BINs:** BOLD:AAC3780

Sequences of *N. dubitana* form in a single BIN (BOLD:AAC3780) with a shallow split between North American representatives (i.e., British Columbia and Washington) and European specimens (i.e., Austria, Finland, Germany, Norway, Italy, United Kingdom); however, specimens from Ontario, Newfoundland, Labrador, and Nova Scotia appear more similar to European specimens than to other North American specimens. 

*Neocochylis* was proposed by Razowski [66] as a subgenus of *Cochylis*, with the type species *Conchylis calavrytana* Rebel, 1906. It later was synonymized with *Cochylis* by Razowski [67], and recently redefined and elevated to generic status by Brown et al. [68], who included *dubitana* in the genus.

*Neocochylis dubitana* is assumed to be Holarctic in distribution [50,60], ranging throughout much of Europe to Asia (China) and throughout much of northern North America. Razowski [60] reported it from Quebec, Alberta, and British Columbia in Canada, and we have examined specimens from Maine in the Northeast, and from Oregon, California, Colorado, Montana, South Dakota, and Wyoming in the West.

***Eugnosta argyroplaca* (Meyrick, 1931)**

**Previous status:** Nearctic (based on previous checklists)

**Revised status:** Palearctic

**Supported by DNA in BOLD:** No (this species not present in BOLD)

**BIN**: N/A

*Eugnosta argyroplaca* is not represented in BOLD. Brown [69] provided the following summary of the status of this species. *Euxanthis argyroplaca* Meyrick, 1931 was described from a single male from “Usgent, Arizona,” U.S.A., but it has not been collected since. Because of its U.S. type locality, the species has long been cited in checklists and catalogs of North American Tortricidae. The male holotype is deposited in the Natural History Museum, London, and the adult and its genitalia were illustrated by Clarke [70].

The species resided in *Euxanthis* until Powell [71] transferred it to *Carolella* Busck. Pogue [64] subsequently transferred it to *Eugnosta* Hübner in his unpublished Ph.D. dissertation. Because *Eugnosta* and *Carolella* are now considered synonyms [68], with *Eugnosta* being the senior synonym, the generic assignment of the species has stabilized. Nonetheless, in a recent list of the Cochylini (Cochylina) of the U.S. and Canada, Metzler and Brown [72] incorrectly returned it to “Cochylini unplaced” without comment. Based on the male genitalia of the holotype, the species is unquestionably assigned correctly to *Eugnosta*. However, there is some doubt regarding the geographic origin of the holotype. In facies, it is similar to several Palaearctic species of *Eugnosta* (e.g., *E. lathoniana*, *E. hydragyrana*, *E. medvedevi*, and *E. magnificana*), with a forewing pattern that features extensive areas of silvery white (e.g., [67]).

This forewing pattern is not known to occur in New World *Eugnosta*. As mentioned previously, the holotype is the only North American record of the species, and the type-locality of “Usgent” could not be located in any map or gazetteer. Although it is possible that “Usgent” is the collector, in all comparable descriptions in Meyrick’s Exotic Microlepidoptera, collectors are given in italics and in parentheses. These lines of evidence seem to suggest that *E. argyroplaca* is a Palearctic species with incorrect collecting information associated with the type.

***Eugnosta californica* Razowski, 1986**

**Previous status:** Present in the U.S. (no status listed in Pohl et al. [28])

**Revised status:** Only recorded from Mexico

**Supported by DNA in BOLD:** No (this species not present in BOLD)

**BINs:** N/A

There are no barcode data for this species. Described from Baja California, Mexico, it may be present in southern California. However, among the large number of specimens of *Eugnosta* we have examined, no U.S. or Canadian specimens match the description and/or illustrations provided by Razowski [73].

***Eugnosta chemsakiana* (Razowski, 1986)**

**Previous status:** Present in the U.S. (no status listed in Pohl et al. [28])

**Revised status:** Only recorded from Mexico

**Supported by DNA in BOLD:** No (this species not present in BOLD)

**BINs:** N/A

There are no barcode data for this species. This species was described from Durango, Mexico, far south of the U.S. border. Among the large number of specimens of *Eugnosta* we have examined, no U.S. or Canadian specimens match the description and/or illustrations provided by Razowski [73].

***Phtheochroa vulneratana* (Zetterstedt, 1839)**

**Previous status:** Holarctic

**Revised status:***Phtheochroa vulneratana* (Palearctic); North American cryptic species (Nearctic)

**Supported by DNA in BOLD:** Yes

**BINs:** BOLD:AAD9720 (Europe); BOLD:AAD8609 (North America)

Sequences of *P. vulneratana* are represented by two BINs, one of which (BOLD:AAD9720) includes only Palearctic specimens (Finland, Italy, Norway, Switzerland) and the other (BOLD:AAD8609) only Nearctic specimens (Canada and U.S.). Powell [74] first proposed that *P. vulneratana* is a Holarctic boreal species, reporting it from Alaska and Colorado; perhaps the earliest record is in the USNM from Colorado, identified as *P. vulneratana* by Jack Clarke in 1967. However, the two BINs containing *P. vulneratana* in BOLD are separated geographically and by >2% (p-dist). Hence, barcode data strongly support the hypothesis that Nearctic and Palearctic specimens represent two different species.

According to Razowski [50,67], *P. vulneratana* ranges from Scandinavia south through the highest mountains of central and southern Europe (e.g., Switzerland, Austria, Italy) and east through the Balkan, Pamir, and Altai mountains, through Siberia, Mongolia, and Amur Territory, to Japan. We have seen North American records identifying this species from British Columbia and Alberta south through the Rocky Mountains to Colorado, at elevations ranging from about 4500 to 12,000 feet; however, it is likely these represent a different taxon.

***Rolandylis maiana* (Kearfott, 1907)**

**Previous status:** Holarctic?

**Revised status:** Nearctic

**Supported by DNA in BOLD:** Yes

**BINs:** BOLD:AAM0777

The only sequences of *R. maiana* in BOLD are from specimens collected in Ontario and are placed in a single BIN (BOLD:AAM0777), along with specimens identified as *R. fusca*, with the latter likely being a misidentification.

Gibeaux [75] described the genus *Rolandylis* for the species *R. catalonica* Gibeaux, 1985. However, there is no question that *catalonica* is conspecific with the North American species *maiana* Kearfott, 1907. Hence, Gibeaux’s specimen (from France) was either mislabeled or represents an inadvertent introduction of this species to Europe. The former seems more likely.

***Spinipogon harmozones* Razowski, 1986**

**Previous status:** Not recorded from the U.S. (in Pohl et al. [28] as *S. thes*)

**Revised status:** Recorded from Texas

**Supported by DNA in BOLD:** No (this species not present in BOLD)

**BINs:** N/A

Pohl et al. [28] listed *Spinipogon thes* Razowski and Becker, 1983 as present in North America. This record is based on a specimen in the USNM collected in Cameron Co., Texas and identified as *S. thes* (type locality Santa Catarina, Brazil). Upon further examination, the specimen in the USNM from Texas is almost certainly *S. harmozones* (type locality Nuevo Leon, Mexico). This is the first record of this species from the U.S. There are no DNA barcode data in BOLD for this taxon (the Texas specimen was submitted but sequencing failed).

***Thyraylia nana* (Haworth, [1811])**

**Previous status:** Holarctic

**Revised status:** Holarctic or Introduced?

**Supported by DNA in BOLD:** Yes

**BINs:** BOLD:ABY5998 (Europe, Canada); BOLD:AAB3573 (North America)

Sequences of *T. nana* are placed in two BINs in BOLD: BOLD:ABY5998 contains specimens from Europe (Austria, Finland, Germany, Norway, United Kingdom) and Canada (Nova Scotia, Quebec), and BOLD:AAB3573 contains specimens from North America (Canada and U.S.). These BINs form a single cluster and likely represent a single taxon. In the Palearctic, this species is known from Europe, Asia Minor, Siberia, and the Russian Far East [50]. In North America, this species is found in the northeastern U.S., south to Tennessee, and west to Minnesota. This species has traditionally been treated as Holarctic; however, because it is not recorded near Beringia or anywhere in western North America, it may be introduced.

***Eulia ministrana* (Linnaeus, 1758)**

**Previous status:** Holarctic

**Revised status:** No change

**Supported by DNA in BOLD:** Yes

**BINs:** BOLD:AAA7315

All of the sequences for *E. ministrana* fall into a single BIN: BOLD:AAA7315. There is a substantial amount of sequence variation, with sequences from several localities forming distinct subclusters, but there is no clear separation between North American and European specimens. This appears to be a single, although genetically variable, Holarctic species.

As currently defined, *Eulia* is monotypic and Holarctic in distribution, ranging from Great Britain across northern Europe and northern Asia to Siberia, and south to Japan [76,77,78]. In North America, it has been recorded from Canada (Alberta, British Columbia, Ontario, Quebec, New Brunswick, Northwest Territories, and Nova Scotia) and the northern United States (Alaska, Washington, Oregon, Idaho, Utah, Pennsylvania, New York, New Hampshire, Maine), ranging south in the eastern U.S. to Virginia, Tennessee, and North Carolina.

***Pandemis cerasana* (Hübner, 1786)**

**Previous status:** Introduced from Eurasia

**Revised status:** No change

**Supported by DNA in BOLD:** Yes

**BINs:** BOLD:AAA3660

Sequences of *P. cerasana* form a single BIN (BOLD:AAA3660), with specimens from North America (British Columbia) sharing nearly identical sequences with those from Europe (Austria, Belgium, the Czech Republic, Finland, Germany, Italy, The Netherlands, and the United Kingdom). The earliest record of *P. cerasana* from North America is from Victoria, British Columbia in 1964 [79]. Mutuura [80] was apparently unaware of Evans’ report (using the junior synonym *P. ribeana*), and stated that the earliest introduction of *P. cerasana* to British Columbia was in 1965. This and the next taxon are common, widespread pest species.

***Pandemis heparana* ([Denis and Schiffermüller], 1775)**

**Previous status:** Introduced from Eurasia

**Revised status:** No change

**Supported by DNA in BOLD:** Yes

**BINs:** BOLD:AAA9254 (Europe, North America); BOLD:ACM3812 (Japan)

Similar to *P. cerasana*, most sequences of *P. heparana* form a single BIN: BOLD:AAA9254, with little sequence variation. Europe specimens are from Austria, the Czech Republic, Finland, Germany, Italy, The Netherlands, Norway, and the United Kingdom. These specimens share identical sequences with those from British Columbia and Washington. There are three specimens identified as *P. heparana* from Japan that are assigned to a separate BIN (BOLD:ACM3812). At least one of these appears to be correctly identified, and *P. heparana* has been recorded from Japan. The two BINs are only 1.65% different (p-dist); thus, this could simply represent geographic variation. More sequence data from Asia are needed to evaluate the status of populations in this region.

*Pandemis heparana* was first discovered in British Columbia in 1978 [80]. This species was reared from larvae collected on apple, crabapple, pear, plum, and species of *Prunus*, *Crataegus*, *Lonicera*, *Rubus*, *Vaccinium*, and *Spiraea* from several locations in the Lower Fraser Valley, British Columbia [81].

***Argyrotaenia franciscana* (Walsingham, 1879)**

**Previous status:** Nearctic (“Introduced” in Pohl et al. [28])

**Revised status:** Nearctic, expanded geographic and host range

**Supported by DNA in BOLD:** Yes

**BINs:** BOLD:AAA9216 (California, Washington, British Columbia); BOLD:ABZ7843 (California, Washington, British Columbia)

Sequences of specimens identified as *A. franciscana* (and the junior synonym *A. citrana*) fall into two BINs, BOLD:AAA9216 and BOLD:ABZ7843, separated by a distance of 1.69% (p-dist). The two BINs form a single cluster and do not appear to have any taxonomic-level significance. Landry et al. [82] performed a phylogenetic analysis of *A. franciscana*, *A. citrana*, *A. franciscana insulana*, and *A. isolatissima* using a larger segment of COI and determined that these taxa form a single monophyletic clade that should be considered a single species. They did, however, find a relatively high amount of variation (3.8% p-dist) in this group. Rubinoff and Powell [83] reanalyzed these same populations and determined that *A. isolatissima* was indeed a valid species separate from the *A. franciscana* complex.

There are several reports of a significant range (and associated host) expansion for *A. citrana*; these are summarized by Powell [57]. It is likely that much of these data is obscured by the uncertain taxonomy in this group, as *A. citrana* and *A. franciscana* were treated as separate species until Landry et al. [82]. For instance, Powell [57] states that *A. citrana* was not found in the San Francisco Bay area before 1910, yet this is the type locality in which *A. franciscana* which was collected by Walsingham in 1871. It does appear that the range of *A. franciscana* to the north and its associated “pest status” did increase at some point, with reports of *A. citrana* in the fruit-growing regions of Oregon and Washington as early as 1930 [57]. Reports of this species in Europe and Florida are erroneous [57,84].

***Choristoneura albaniana* (Walker, 1863)**

**Previous status:** Holarctic

**Revised status:** No change

**Supported by DNA in BOLD:** Yes

**BINs:** BOLD:AAB3030

Sequences of *C. albaniana* fall into a single BIN: BOLD:AAB3030, with representatives from Ontario, Manitoba, British Columbia, and Finland. The specimens from Finland cluster separately from the Canadian specimens, but overall genetic variation is low. Hence, it appears that this is a single Holarctic species with some minor geographic variability.

***Archips oporana* (Linnaeus, 1758)**

**Previous status:** Introduced from Eurasia

**Revised status:** No change

**Supported by DNA in BOLD:** Yes

**BINs:** BOLD:AAD6710

Sequences from specimens of *A. oporana* from Europe (Austria, Finland, Germany, Italy, The Netherlands, Norway), Asia (Japan, South Korea), and North America (Canada) are represented in BOLD. All sequences are placed in BIN BOLD:AAD6710, with little to no variation; the majority of specimens share identical barcode sequences. One sequence from Japan is labeled as *A. audax*, but this is likely a misidentification. Freeman [85] indicates that this species was collected from two different locations in Canada: Vancouver, British Columbia in 1937, and Font Hill, Ontario. Pohl et al. [46] were unable to locate these specimens for verification and suggest that if this species was found in Canada at one time, it did not establish. However, BOLD contains sequences from three specimens collected in Ontario from 2013–2017, indicating this species is still present in Canada.

***Archips xylosteana* (Linnaeus, 1758)**

**Previous status:** Introduced from Eurasia

**Revised status:** No change

**Supported by DNA in BOLD:** No (no specimens from North America in BOLD)

**BINs:** BOLD:AAC0366

Sequences of *A. xylosteana* in BOLD are from specimens originating in Europe and northern Africa. These form a single cluster and are placed in the BIN BOLD:AAC0366. Although no sequences from North America are represented in BOLD, there is little doubt that this species was introduced recently to Canada. Hoebeke et al. [86] reported specimens of *A. xylosteana* collected in 2005 and 2006 from the St. John’s area of the Avalon Peninsula in Newfoundland.

***Archips rosana* (Linnaeus, 1758)**

**Previous status:** Introduced from Eurasia

**Revised status:** No change

**Supported by DNA in BOLD:** Yes

**BINs:** BOLD:AAB9404

*Archips rosana* is represented in BOLD by specimens from Europe (Austria, Finland, Germany, Italy, and Norway) and North America (British Columbia and Washington). All sequences form a single BIN, BOLD:AAB9404, with several specimens labeled as *A. grisea*, which are assumed to be misidentifications. This species was first reported in North America, likely arriving in 1889, by Comstock and Slingerland [87] from Albany, New York. It was found in Canada for the first time in 1919, both in Victoria, British Columbia, and Nova Scotia [41]. It has since spread to other locations in the East, including Connecticut, New Brunswick, Nova Scotia, Ontario, Pennsylvania, Prince Edward Island, and Quebec [46,85,88], and other locations in the West, including Alaska, Alberta, Oregon, and Washington [46,88].

***Archips podana* (Scopoli, 1763)**

**Previous status:** Introduced from Eurasia

**Revised status:** No change

**Supported by DNA in BOLD:** Yes

**BINs:** BOLD:AAB5839

The majority of sequences of *A. podana* are from European specimens, which form a single BIN, BOLD:AAB5839, with little genetic variation. However, two specimens from Canada (British Columbia) share identical barcode sequences with several of the European specimens. Freeman [85] reported that *A. podana* was first recorded in North America from British Columbia in 1937; however, these records were apparently confused with those of *A. oporana* [89]. The first confirmed record of *A. podana* in North America is from the lower Fraser River Valley in British Columbia in 1988 [42]. LaGasa et al. [89] provided details of surveys for this species in Whatcom County, Washington in the early 2000s, representing the first records for the U.S. The report of *A. podana* from Ontario by Belton [42] is an error [46].

***Archips fuscocupreanus* Walsingham, 1900**

**Previous status:** Introduced from Eurasia

**Revised status:** No change

**Supported by DNA in BOLD:** No (no specimens from outside North America in BOLD)

**BINs:** BOLD:AAD6614

The only specimens of *A. fuscocupreanus* represented in BOLD are from Washington, which fall into a single BIN, BOLD:AAD6614, and form two clusters with little genetic variation. *Archips fuscocupreanus* was first discovered in North America in Connecticut, where it has been present since at least 1982 [90]. Maier [91] determined that this species was widespread in the northeastern U.S. (Connecticut, Massachusetts, New Jersey, New York, and Rhode Island). He also discovered two specimens in the collection of the USNM from Washington collected in 1995 [91].

***Cacoecimorpha pronubana* (Hübner, 1822)**

**Previous status:** Introduced, likely from Europe

**Revised status:** No change

**Supported by DNA in BOLD:** No (no specimens from North America in BOLD)

**BINs:** BOLD:AAD3477 (Europe); BOLD:AAL5782 (Spain)

Sequences from specimens of *C. pronubana* are resolved into two separate BINs: BOLD:AAD3477 contains specimens from Europe (France, Germany, The Netherlands, Norway, United Kingdom) and BOLD:AAL5782 contains specimens from Spain. These two BINs are separated by >3.0% (p-dist). There are no phenotypical differences in the specimens that would provide evidence that two species are involved, although it is possible that specimens from Spain represent a cryptic species. No specimens from North America are represented in BOLD, although some have been sequenced in other facilities.

*Cacoecimorpha pronubana* was found in Oregon in 1964 [92]. It has since spread to Washington, with the first record in 1974; surveys in the late 1990s revealed that it was widespread in western Washington [93]. In 2011, *C. pronubana* larvae were found in California feeding on *Daphne* (Thymelaeaceae), and in 2013 an infestation of *C. pronubana* was found in the Denver Zoo in Denver, Colorado [94].

***Dichelia histrionana* (Frölich, 1828)**

**Previous status:** Introduced from Europe

**Revised status:** No change

**Supported by DNA in BOLD:** Yes

**BINs:** BOLD:AAC2478 (Europe, Canada); BOLD:ACF5563 (Europe)

Sequences of *D. histrionana* fall into two BINs: BOLD:AAC2478 includes specimens from Europe (Austria, Denmark, Italy) and Canada; and BOLD:ACF5563 contains specimens from Europe (Austria, Finland, Germany, Norway). The two BINs are separated by only 1% (p-dist), and both cluster together, suggesting that they are the same species. DeWaard et al. [95] first discovered this species in North America while conducting a DNA barcoding study of moth species found in Stanley Park, Vancouver, Canada.

***Clepsis spectrana* (Treitschke, 1830)**

**Previous status:** Introduced from Europe

**Revised status:** No change

**Supported by DNA in BOLD:** Yes

**BINs:** BOLD:AAC1795

Sequences of *C. spectrana* are placed in a single BIN in BOLD (BOLD:AAC1795), with specimens from Europe (Finland, Germany, Italy, The Netherlands, Norway, United Kingdom) and Canada (British Columbia, Quebec). The earliest record in North America is a single specimen collected in British Columbia in 1950 [96]. This species was rediscovered feeding on raspberry, currant, spruce, and cedar in the early 1990s in British Columbia, and the first U.S. record was reported from Washington in 1997 [97]. Razowski [98] records the Palearctic distribution for this species as Central, Northern, and East Europe.

***Clepsis danilevskyi* Kostiuk, 1973**

**Previous status:** Holarctic

**Revised status:** No change

**Supported by DNA in BOLD:** No (this species not present in BOLD)

**BINs:** N/A

This species is not represented in BOLD. *Clepsis danilevskyi* is present in Alaska and the Yukon [46] in the Nearctic, and in the Altai and Polar Ural Mountains in the Palearctic [50]. *Clepsis firthana*, described by Mutuura [99] from Canada, is considered a junior synonym of *C. danilevskyi*.

***Clepsis consimilana* (Hübner, 1822)**

**Previous status:** Introduced from Eurasia

**Revised status:** No change

**Supported by DNA in BOLD:** Yes

**BINs:** BOLD:AAC4212

Specimens of *C. consimilana* are placed in a single BIN (BOLD:AAC4212) in BOLD with representatives from Europe (Austria, Denmark, Germany, Italy, United Kingdom) and North America (British Columbia, Washington). Zlatkov and Huemer [100] revised a group of European *Clepsis*, including this species, and determined the following regarding BOLD:AAC4212: “The intraspecific average of the barcode region is 0.34%, the maximum distance 1.08% (p-dist) (*n* = 29). The minimum distance to the nearest neighbour, *Clepsis eatoniana*, is 2.25%.”

Several other single specimens labeled as *C. consimilana* in other BINs are likely misidentified. This species was first reported from North America by Klots [36] (as *C. unifasciana*), who identified two specimens from Long Island, New York collected in 1939. Powell [101] reported it from Oregon, and Dang et al. [96] reported the first records from British Columbia. The distribution of *C. consimilana* in the Palearctic includes Europe, Asia Minor, Syria, European Russia, and West Africa to Lebanon; it had been reported as introduced to Madagascar [100], but that population may represent a different species.

***Clepsis moeschleriana* (Wocke, 1862)**

**Previous status:** Holarctic

**Revised status:** No change

**Supported by DNA in BOLD:** No (no specimens from outside North America in BOLD)

**BINs:** BOLD:AAB4040

All of the sequences of *C. moeschleriana* are placed in a single BIN in BOLD (BOLD:AAB4040), with specimens from Canada and Alaska. In the Palearctic, this species has been recorded from the Polar Ural Mountains in Russia and the Altai Mountains in Central Asia [50]. In North America, this species is recorded from Labrador, across Canada to Alaska [46], and from a few unverified records in other U.S. states.

***Clepsis illustrana* (Krogerus, 1936)**

**Previous status:** Holarctic (no specific status listed in Pohl et al. [28])

**Revised status:** Holarctic?

**Supported by DNA in BOLD:** No (sequence clustering is ambiguous)

**BINs:** BOLD:AAF4488 (Finland); BOLD:ACL6434 (Canada)

Sequences of *C. illustrana* in BOLD form two separate BINs: BOLD:AAF4488 contains three specimens from Finland, and BOLD:ACL6434 contains three specimens from Canada (Manitoba, Ontario, Yukon Territory). The two BINs cluster together with less than 2% (p-dist) overall variation; hence, the two BINs could represent closely related species or variation between two isolated populations of the same species: one in the Palaearctic and one in the Nearctic. Ferris et al. [102] first recorded this species from North America. We treat it here as tentatively Holarctic pending the sequencing and examination of additional specimens.

***Epiphyas postvittana* (Walker, 1863)**

**Previous status:** Introduced from Australia

**Revised status:** No change

**Supported by DNA in BOLD:** Yes

**BINs:** BOLD:AAB0679

Nearly 1600 sequences of *E. postvittana* are present in BOLD, and all are assigned to a single BIN (BOLD:AAB0679). This species is native to Australia and has been introduced to Tasmania, New Zealand, Hawaii, the United Kingdom, and the Azores, with isolated records from other countries in Europe, including The Netherlands. This species was first detected in California in 2006 [12], and it has since become established and common along most of the coastal counties from Mendocino south to San Diego. Genetic studies have determined that there were likely two separate introductions of this species into California, with Australia being the most likely origin [103].

***Ditula angustiorana* (Haworth, [1811])**

**Previous status:** Introduced from Europe

**Revised status:** No change

**Supported by DNA in BOLD:** Yes

**BINs:** BOLD:AAA8699

*Ditula angustiorana* is represented in BOLD by specimens from Europe (Denmark, Germany, Italy, The Netherlands, Norway, and United Kingdom) and Canada, and all are placed in a single BIN (BOLD:AAA8699). The majority of DNA barcodes for this species are nearly identical. This species was first recorded in North America from Victoria, British Columbia in 1924 [41]. It is currently present along the West Coast from Vancouver Island south to the San Francisco Bay Area.

***Sparganothis rubicundana* (Herrich-Schäffer, 1856)**

**Previous status:** Holarctic?

**Revised status:** Holarctic

**Supported by DNA in BOLD:** No (no specimens from North America in BOLD)

**BINs:** BOLD:AAF4352

The only specimens of *S. rubicundana* in BOLD are from Europe (Finland and Norway) and are placed in a single BIN (BOLD:AAF4352). Powell [71] synonymized *S. hudsoniana* Freeman, 1940, from Manitoba with the Palearctic *S. rubicundana*. Powell and Brown [104] followed this treatment and recorded additional specimens from Alaska and Nunavut. Pohl et al. [46] added the Northwest Territories and Ontario to the distribution for this species. In the absence of DNA evidence to the contrary, we assume that this is a Holarctic species.

***Sparganothis praecana* (Kennel, 1900)**

**Previous status:** Holarctic

**Revised status:** No change

**Supported by DNA in BOLD:** No (no specimens from North America in BOLD)

**BINs:** BOLD:AAE8754

The only specimens of *S. praecana* present in BOLD are from Europe (Austria, Finland, Norway) and are placed in a single BIN (BOLD:AAE8754). Powell and Brown [104] documented this presumably northern Holarctic species from the Yukon and Northwest Territories.

***Platynota stultana* Walsingham, 1884**

**Previous status:** Nearctic (Pohl et al. [28] state “Introduced,” likely referring to other regions of North America)

**Revised status:** Nearctic, expanded geographic and host range; introduced to Europe

**Supported by DNA in BOLD:** Yes

**BINs:** BOLD:AAB6120 (California, Florida); BOLD:AAC5877 (near San Diego, California)

Specimens identified as *P. stultana* in BOLD are placed in two BINs that are separated by >3% (p-dist): BOLD:AAB6120 contains specimens from California and Florida, and BOLD:AAC5877 contains specimens collected near San Diego, California. It is possible that the San Diego population represents a cryptic species.

Powell and Brown [104] provide a detailed account for this species. A native of Mexico and possibly the southwestern U.S., *P. stultana* was introduced into California sometime around 1898. It greatly expanded its geographic range northwards in the 1960s. At the same time, it expanded its host range onto many nonnative plants [105], including important crops, and has now become an important pest of grapes and greenhouse plants [94]. It was reported from Florida in the 1940s, where it is apparently established [106]. *Platynota stultana* has been recorded from California, Arizona, Hawaii, Texas, Florida, Mexico, and there are sporadic records from other U.S. states, although it is probably not established elsewhere. This species was reported on peppers in Spain in 2009, and Groenen and Baixeras [107] documented its presence in that country as early as 2005.

**Subfamily OLETHREUTINAE**

***Endothenia quadrimaculana* (Haworth, [1811])**

**Previous status:** Introduced from Europe

**Revised status:** No change

**Supported by DNA in BOLD:** No (no specimens from North America in BOLD)

**BINs:** BOLD:AAD4973

Sequences of *E. quadrimaculana* in BOLD are placed in a single BIN, BOLD:AAD4973, with representatives from Austria, Finland, Germany, The Netherlands, and Norway. Most historical references to *E. quadrimaculana* in North American refer to *E. nubilana* (BOLD:AAC1943); these names were synonyms until Miller [108] elevated *nubilana* to species status, recognizing it as a Nearctic species. Pohl et al. [46] reported the first introduction of the European *E. quadrimaculana* into North America, and they state it is established in Ontario and Quebec.

***Endothenia gentianaeana* (Hübner, [1799])**

**Previous status:** Introduced

**Revised status:** Palearctic (misidentification)

**Supported by DNA in BOLD:** Yes

**BINs:** BOLD:ACE6355

Sequences of *E. gentianaeana* are placed in a single BIN, BOLD:ACE6355, with specimens from Austria, Finland, Germany, and the United Kingdom. This species has been proposed as a biocontrol agent for teasel [109]. Beebe [110] reported that it was present in Michigan; however, Miller [108] determined that the specimens to which Beebe referred were misidentified *E. hebesana*.

***Tia enervana* (Erschoff, 1877)**

**Previous status:** Holarctic

**Revised status:** No change

**Supported by DNA in BOLD:** No (sequencing of specimens in BOLD failed)

**BINs:** N/A

Specimens from Alberta and Alaska are included in BOLD, although sequencing failed for all of them; hence, there are no DNA data. Jalava and Miller [111] confirmed the synonymy of the European taxon *Penthina enervana* Erschoff, 1877 with the North American taxon *Argyroploce vulgana* McDunnough, 1922 based on morphology.

***Bactra lancealana* (Hübner, [1799])**

**Previous status:** Holarctic

**Revised status:** No change

**Supported by DNA in BOLD:** Yes

**BINs:** BOLD:AAB8686

Sequences from specimens identified as *B. lancealana* are placed in a single BIN in BOLD (BOLD:AAB8686). This includes examples from Europe (Austria, Finland, Germany, Italy, The Netherlands, Norway, United Kingdom) as well as Canada (Manitoba, Quebec, Northwest Territories). This BIN also includes a single cluster of specimens identified as *B. robustana*; it is unknown if these are misidentifications or if these two taxa share similar barcodes. *Bactra lancealana* is the most common species of *Bactra* in Central Europe, and misidentifications are common in this genus because many species are superficially similar.

***Bactra furfurana* (Haworth, [1811])**

**Previous status:** Holarctic

**Revised status:** No change

**Supported by DNA in BOLD:** Yes

**BINs:** BOLD:AAA7927 (Canada, U.S.); BOLD:ACE7672 (Washington); BOLD:ABZ2466 (Finland, Norway, United Kingdom)

Sequences of *B. furfurana* are placed in three BINs in BOLD: BOLD:AAA7927 contains specimens from Canada and the U.S.; BOLD:ACE7672 contains specimens from Washington; and BOLD:ABZ2466 contains specimens from Finland, Norway, and the United Kingdom. Although it is possible that these different BINs represent different species, all three are resolved in a single cluster, and there is no further evidence that more than one taxon is represented in the samples. This is a common and widespread species.

***Bactra priapeia* Heinrich, 1923**

**Previous status:** Neotropical (listed as “Holarctic?” in Pohl et al. [28], likely a mistake)

**Revised status:** Neotropical

**Supported by DNA in BOLD:** Yes

**BINs:** BOLD:AAA0293

Specimens identified as *B. priapeia* from Florida are placed in a single BIN (BOLD:AAA0293) with specimens identified as *B. verutana* from Costa Rica and a single unidentified specimen from French Guiana. The Costa Rica “*B. verutana*” specimens are likely misidentified, as this BIN is distant from the main *B. verutana* BIN (BOLD:AAB4642) with verified specimens of that species. Heinrich [112] described this species from Louisiana, with a distribution of Texas, Louisiana, Florida, and Panama. Powell [113] states that this species is likely introduced to coastal southern California, and we examined specimens collected near San Diego.

***Lobesiodes euphorbiana* (Freyer, 1842)**

**Previous status:** Introduced from Europe

**Revised status:** No change

**Supported by DNA in BOLD:** No (this species not present in BOLD)

**BINs:** N/A

This species is not represented in BOLD. *Lobesiodes euphorbiana* was released into several Canadian provinces from Europe for the control of leafy spurge. It was established in Manitoba and Ontario [114], but it is not known if introductions in other areas were successful [46].

***Lobesia bicinctana* (Duponchel, 1843)**

**Previous status:** Holarctic

**Revised status:***Lobesia bicinctana* (Palearctic); *Lobesia spiraeae* (McDunnough, 1938), **stat. rev.** (Nearctic)

**Supported by DNA in BOLD:** Yes

**BINs:** BOLD:AAD7409 (Finland, Norway; *L. bicinctana*); BOLD:AAD7408 (Newfoundland, Quebec; *L. spiraeae*)

In BOLD, specimens of *L. bicinctana* are placed in two BINs that are separated by 2.93% (p-dist): BOLD:AAD7409 contains specimens from Finland and Norway, and BOLD:AAD7408 contains specimens from Newfoundland and Quebec. McDunnough [115] described *Lobesia bicinctana spiraeae* from Nova Scotia. Royals et al. [22] found that the Canadian populations of *L. bicinctana* are well-supported as being a separate taxon from the *L. bicinctana* populations in Europe (but this taxon was not formally elevated in Royals et al. [22]). Based on this evidence, we elevate *L. spiraeae* (McDunnough, 1938), stat. rev., to species status. This is the only species of *Lobesia* currently present in North America [22].

***Lobesia botrana* ([Denis and Schiffermüller], 1775)**

**Previous status:** Introduced

**Revised status:** Eradicated from the U.S. in 2016

**Supported by DNA in BOLD:** Yes

**BINs:** BOLD:ACH2178

Specimens of *L. botrana* in BOLD are placed in a single BIN: BOLD:ACH2178. This species was first discovered in North America when larvae were found on grape clusters in Napa Valley, California in 2008 [13]. This discovery prompted an extensive survey and control program in the wine-growing regions of California. The United States Department of Agriculture (USDA) and the California Department of Agriculture (CDFA) announced the moth’s eradication status on August 18, 2016, two years after the last adult specimens were captured. This species was also introduced into Argentina and Chile [13], where it remains a serious pest.

***Apotomis capreana* (Hübner, [1817])**

**Previous status:** Holarctic

**Revised status:** No change; possible cryptic species in North America

**Supported by DNA in BOLD:** No (misidentifications and/or DNA barcodes do not separate species)

**BINs:** BOLD:ABZ6958 (Europe and Canada); BOLD:ACE4055 (Canada); BOLD:AAA4846 (Canada)

*Apotomis capreana* is represented in BOLD with sequences in three BINs: BOLD:ABZ6958 contains specimens from Europe and Canada identified as *A. capreana* and a variety of other *Apotomis* species; BOLD:ACE4055 contains specimens from Canada and Alaska that group into two main clusters, one identified as *A. removana* and the other identified as *A. capreana*; and BOLD:AAA4846 contains specimens from Canada identified as *A. capreana* and a variety of other *Apotomis* species.

Identification of Apotomis to species is difficult, even with genitalic dissection [116]. As such, most of the identifications in BOLD require confirmation before any conclusions can be made based on sequence clustering, BIN assignment, etc. Some BINs, such as BOLD:ABZ6958, contain approximately 250 specimens identified as 10+ different species of *Apotomis*. It is also possible that DNA barcodes cannot convincingly separate the species in this genus.

***Apotomis infida* (Heinrich, 1926)**

**Previous status:** Holarctic

**Revised status:** No change; possible cryptic species in North America

**Supported by DNA in BOLD:** No (misidentifications and/or DNA barcodes do not separate species)

**BINs:** BOLD:ACF3687 (Europe, Canada); BOLD:AAA4846 (Canada)

Specimens identified as *A. infida* in BOLD are placed into two BINs: BOLD:ACF3687 contains specimens identified as *A. infida* and *A. moestana* from Europe and Yukon Territory, and BOLD:AAA4846 contains specimens from Canada identified as *A. infida* and a variety of other *Apotomis* species. There is currently insufficient data in BOLD to make any definitive conclusions regarding this species. See the previous taxon for more details.

***Cymolomia hartigiana* (Ratzeburg, 1840)**

**Previous status:** Introduced from Europe

**Revised status:** No change

**Supported by DNA in BOLD:** Yes

**BINs:** BOLD:AAE3063 (Europe, U.S.); BOLD:ACF2297 (Finland, Norway)

Sequences of *C. hartigiana* in BOLD fall into two BINs: BOLD:AAE3063 contains specimens from Austria, Germany, and New York), and BOLD:ACF2297 contains specimens from Finland and Norway. Both BINs form a single cluster and are separated by 1.65% (p-dist). We assume that these BINs represent geographical variation and not separate taxa.

McGuinness and Brown [117] reported *Cymolomia hartigiana* from Long Island, New York based on eight specimens collected during survey work conducted by McGuinness from 2007 to 2016. This species is undoubtedly a recent arrival in North America, and given the range of collection years (2007–2016), it is almost certainly established in New York.

***Orthotaenia undulana* ([Denis and Schiffermüller], 1775)**

**Previous status:** Holarctic (no designation in Pohl et al. [28])

**Revised status:** Holarctic, possible cryptic species in North America

**Supported by DNA in BOLD:** Yes

**BINs:** BOLD:AAB4021 (Europe, Alaska; *O. undulana*); BOLD:AAA8540 (Canada, U.S.; cryptic sp.); BOLD:AAB4022 (Pacific Northwest; possibly *Olethreutes deprecatoria*)

Sequences of specimens identified as *O. undulana* in BOLD are placed in three BINs: BOLD:AAA8540 contains specimens from Canada (Alberta, British Columbia, Manitoba, New Brunswick, Newfoundland, Nova Scotia, Ontario, Prince Edward Island, Quebec, Saskatchewan) and the U.S. (California, Ohio, Washington); BOLD:AAB4021 contains specimens from Europe (Austria, Finland, Germany, Italy, The Netherlands, Norway, United Kingdom) and Alaska; and BOLD:AAB4022 contains specimens from British Columbia, Oregon, and Washington.

There is considerable confusion regarding the identity of specimens in these BINs, which are sufficiently separated to assume that they all represent distinct taxa. *Orthotaenia undulana* was described from Austria, and thus, it is likely that BOLD:AAB4021 represents this species. This BIN consists of two main clusters: one with specimens from Alaska, The Netherlands, and Italy, and the other with specimens from across Europe. There is no sequence variation in the first cluster, and minor variation in the second; i.e., the overall variation in the BIN is only 0.62% (p-dist). It is possible that identical sequences in Europe and Alaska could indicate that *O. undulana* is introduced; however, without any direct evidence of such, and with low overall sequence variation, the simplest explanation is that this BIN represents a single Holarctic taxon.

The majority of specimens from Canada and the U.S. that are identified as *O. undulana* in BOLD fall into a separate BIN, BOLD:AAA8540. We noticed subtle differences in both the male and female genitalia between specimens traditionally identified as *O. undulana* from Europe and North America. As such, it is possible that BOLD:AAA8540 represents a cryptic species present only in North America. If so, there are two current junior synonyms of *O. undulana* described from North America that could apply. The oldest is *Sericoris campestrana* Zeller, 1875, described from Maine or Massachusetts; the other species is *Sericoris dilutifuscana* Walsingham, 1879, described from southern Oregon.

The third BIN, BOLD:AAB4022, contains specimens that are clearly not *O. undulana* based on photos of the wing pattern and genitalic dissections. Specimens in this BIN were tentatively identified as the next taxon, *Syricoris lacunana*, based on dissections of specimens from Washington and Oregon. However, Steve Nanz, Editor in Chief of the Moth Photographers Group, has brought to our attention that the male genitalia for specimens in BOLD:AAB4022 are more similar to those of *Olethreutes deprecatoria* Heinrich, 1926, than to *S. lacunana*. This BIN is also well-separated from BOLD:AAC3531 (next taxon), which we believe to represent the true *S. lacunana*. In addition, given the similarity in genitalia of *O. deprecatoria* to *S. lacunana*, it is likely that *O. deprecatoria* would be more properly placed in *Syricoris*.

***Syricoris lacunana* ([Denis and Schiffermüller], 1775)**

**Previous status:** Palearctic

**Revised status:** Holarctic

**Supported by DNA in BOLD:** Yes

**BINs:** BOLD:AAC3531 (Europe, Canada)

In BOLD, sequences of *S. lacunana* (most identified as *Celypha lacunana*) are placed in a single BIN, BOLD:AAC3531, with representatives from Europe (Austria, Denmark, Finland, Germany, Italy, Macedonia, The Netherlands, Norway, Poland, Romania, United Kingdom) and Canada (Alberta, British Columbia, Newfoundland, Nova Scotia, Quebec). A few specimens identified as *S. lacunana* in other BINs, including BOLD:AAB4022 (see previous taxon), are misidentifications.

*Syricoris lacunana* has not been previously recorded from North America. DNA barcodes indicate that this species is present in both eastern and western Canada. Based on its northern distribution in North America, we believe it is likely Holarctic instead of introduced. This species is similar in wing pattern to other taxa in North America (*Olethreutes*, *Celypha*, *Orthotaenia*), so it is not surprising that it could have remained undetected until now.

**NOTE:** Heinrich [25] attempted to resolve the taxonomy of the North American species of “*Olethreutes*” by synonymizing *Argyroploce*, *Phiaris*, *Celypha*, *Orthotaenia*, *Selenodes*, and *Mixodia* with *Olethreutes*, and recognizing *Exartema* as a distinct, closely related genus. *Exartema* was subsequently synonymized with *Olethreutes*, resulting in essentially a single large genus for all North American species formerly included in this array of genera [71]. In Europe, these proposed changes were either short-lived or simply ignored, and the European definition of *Olethreutes* includes only the type (*O. arcuella*) and two additional species. Although Brown [26] made some progress toward resolving discrepancies in the generic assignments between the North American and European faunas: nearly all of the North American species remaining in *Olethreutes* would be assigned to different genera in European treatments, and all of the species listed below under *Olethreutes* are currently assigned to either *Phiaris* or *Argyroploce* in the most recent European checklist [118]. We generally agree with the European treatment, but hesitate to make extensive changes in generic assignment to species that occur in North America until there is a comprehensive revision of the group that includes all of these taxa. Nedoshivina [119] provides the most recent revision of generic concepts in this group, but her work is not particularly relevant to the North American fauna.

***Olethreutes metallicana* (Hübner, 1796)**

**Previous status:** Holarctic

**Revised status:** Holarctic?

**Supported by DNA in BOLD:** No (misidentifications and/or DNA barcodes do not separate species)

**BINs:** BOLD:AAB1043

The BIN BOLD:AAB1043 is divided into several distinct clusters: the first contains specimens identified as *O. metallicana* from Canada and Alaska; the second contains specimens identified as *O. nordeggana* from Canada and *Phiaris obsoletana* (Zetterstedt, 1839) from Finland, Norway, and Russia; and the third cluster contains specimens identified as *Phiaris metallicana* from Europe. There is little genetic variation within each cluster.

Miller [120] synonymized *O. metallicana* with *murina* Packard, 1866 (described from Canada) and *major* Walsingham, 1895 (described from Colorado). He did note a slight difference in the female genitalia, but did not have a large enough sample size to determine if this was significant. Based on the DNA data, it appears that the European and North American populations are potentially different species. However, Nedoshivina [119] includes many records of this species from the Russian Far East, indicating that it may indeed be Holarctic. Additional study is necessary to resolve the status of this species.

***Olethreutes nordeggana* (McDunnough, 1922)**

**Previous status:** Holarctic

**Revised status:** No change

**Supported by DNA in BOLD:** No (misidentifications and/or DNA barcodes do not separate species)

**BINs:** BOLD:AAB1043

Miller and Jalava [121] determined that records of *O. obsoletana* from northeast Siberia were actually misidentified *O. nordeggana*. They state that the two species are very similar but can be separated by differences in the structure and size of the male cornuti. It is not known if the specimens of *Phiaris obsoletana* in BOLD that cluster with *O. nordeggana* are also misidentified, if these are actually the same taxa, or if DNA barcodes are unable to separate these species. In either case, it seems that *O. nordeggana* is likely Holarctic. In Europe, this taxon is currently treated under *Phiaris* [118].

***Olethreutes heinrichana* (McDunnough, 1927)**

**Previous status:** Holarctic

**Revised status:** No change

**Supported by DNA in BOLD:** Yes

**BINs:** BOLD:AAA8541

Specimens of *O. heinrichana* are placed in a single BIN in BOLD (BOLD:AAA8541) with representatives from Canada, Finland, and Norway (European specimens identified as *Phiaris heinrichana*). Jalava and Miller [111] synonymized the previously Nearctic *heinrichana* with the Palearctic *hyperboreana*, creating a Holarctic taxon. The DNA data appear to support this conclusion.

***Olethreutes schulziana* (Fabricius, 1777)**

**Previous status:** Holarctic

**Revised status:** No change

**Supported by DNA in BOLD:** No (misidentifications and/or DNA barcodes do not separate species)

**BINs:** BOLD:AAB9826 (Canada), BOLD:AAB9825 (Greenland and Canada), BOLD:ACF5701 (Europe, Yukon Territory)

There are several BINs in BOLD that contains sequences from specimens identified as various species of *Olethreutes* (primarily North America) and *Phiaris* (primarily Europe). Identification of many of these species is difficult, especially without a genitalic dissection. Thus, it is difficult to know if the inclusion of multiple species in multiple BINs is the result of misidentifications, if some of the taxa are synonyms, or if the DNA barcode data are insufficient for separating these taxa. This is especially true for *O. schulziana* and the next few *Olethreutes* species.

Sequences of *O. schulziana*, *O. turfosana*, *O. septentrionana*, and *O. inquietana* are placed in several BINs in BOLD: BOLD:AAB9826 contains specimens from Canada identified as *O. schulziana* and *O. turfosana*; BOLD:AAB9825 contains specimens from Canada and Greenland identified as *O. schulziana* and *O. inquietana*; and BOLD:ACF5701 contains specimens from Europe and Yukon Territory identified as *P. schulziana*, *P. palustrana*, *P. septentrionana*, *P. turfosana*, and *Phiaris* sp. As stated above, it is difficult to know if many of these specimens are simply misidentified or if they cannot be separated by DNA barcodes. Therefore, the DNA data is currently of limited use for these taxa.

*Olethreutes schulziana* was listed as Holarctic by Lafontaine and Wood [122]. It is not known when it was first discovered in North America, but Pohl et al. [46] list records from most of Canada and Alaska.

***Olethreutes turfosana* (Herrich-Schäffer, 1851)**

**Previous status:** Holarctic

**Revised status:** No change

**Supported by DNA in BOLD:** No (misidentifications and/or DNA barcodes do not separate species)

**BINs:** See summary under *O. schulziana*

Jalava and Miller [111] formally synonymized *Mixodia* ? *intermistana* Clemens, 1865, described from Labrador, with *O. turfosana*, resulting in a Holarctic taxon. This species is currently recorded from Alaska, Maine, and much of Canada. Nedoshivina [119] records this species from much of Russia, including the Far East. In Europe, this taxon is currently treated under *Phiaris* [118].

***Olethreutes septentrionana* (Curtis, 1835)**

**Previous status:** Holarctic

**Revised status:** No change

**Supported by DNA in BOLD:** No (misidentifications and/or DNA barcodes do not separate species)

**BINs:** See summary under *O. schulziana*

*Orthotaenia septentrionana* was described by Curtis in the appendix of Ross’ “Narrative of a second voyage in search of a north-west passage and of a residence in the arctic regions during the years 1829–1833.” Although Heinrich [25] lists the type locality of this species as “Arctic American,” there is nothing in the original description to indicate the origin of the types (“The box contained two specimens of this small Tortrix, which resembles a little the *T. hybridana* of Hübner, pl. 38, fig, 238.”), and the prior species account of *bentleyana* describes specimens collected in England.

Regardless, there are two junior synonyms of *septentrionana* that are described from the Nearctic, and Jalava and Miller [111] synonymized the Palearctic *schaefferana* with *septentrionana*, resulting in a Holarctic taxon. In North America, it is found in Alaska, Maine, and parts of Canada. It is also present in the Russian Far East [119].

***Olethreutes inquietana* (Walker, 1863)**

**Previous status:** Holarctic

**Revised status:** No change

**Supported by DNA in BOLD:** No (misidentifications and/or DNA barcodes do not separate species)

**BINs:** See summary under *O. schulziana*

As with the previous three species, it is difficult to determine the distribution or even taxonomic status of this species. Heinrich [25] included several junior synonyms under *inquietana* that have since been placed under *turfosana*. Currently, *O. inquietana* is the senior synonym of *hepialana* (described from Mongolia), *hoyoshi* (described from Greenland), and *retortimacula* (described from Munku-Sardyk on the border of Mongolia and Russia). In North America, this species is found in Alaska, Maine, and parts of Canada. We assume it is Holarctic pending further analysis of the junior synonyms. In Europe, this taxon is currently treated under *Phiaris* [118].

***Olethreutes mengelana* (Fernald, 1894)**

**Previous status:** Nearctic

**Revised status:** Holarctic

**Supported by DNA in BOLD:** No (no specimens from Europe in BOLD)

**BINs:** BOLD:AAB9942

This species was described from McCormick Bay, Greenland. Miller and Jalava [121] included it in their study of boreal Olethreutini, but concluded that it was restricted to the Nearctic. Aarvik [118] states that it is present in Novaya Zemlya, an archipelago off northern Russia in the Arctic Ocean. This would indicate that it is indeed a Holarctic taxon. In Europe, this species is listed under the combination *Argyroploce mengelana* [118]. There are only six specimens of this species represented in BOLD, all of which were collected from Nunavut, Canada.

***Olethreutes exaridanus* Kuznetsov, 1991**

**Previous status:** Holarctic

**Revised status:** No change

**Supported by DNA in BOLD:** No (this species not present in BOLD)

**BINs:** N/A

This species is currently unrepresented in BOLD. It was known only from Russia until Jalava and Miller [111] reported a specimen from the Ogilvie Mountains in the Yukon, Canada collected in 1985. The type locality is Chukotka in the Russian Far East. According to Nedoshivina [119], this species should likely be placed in *Argyroploce*.

***Olethreutes palustrana* (Lienig and Zeller, 1846)**

**Previous status:** Holarctic

**Revised status:** No change

**Supported by DNA in BOLD:** No (no specimens from North America in BOLD)

**BINs:** BOLD:AAC8201 (Germany, Finland, Italy, Austria, Norway)

Specimens of *O. palustrana* with sequences in BOLD are placed in a single BIN (BOLD:AAC8201) with representatives from Austria, Finland, Germany, Italy, and Norway. Jalava and Miller [111] documented the first North American records for this species with specimens collected at Schrader and Galbraith Lakes in Alaska. This species is widespread in Europe and occurs in far eastern Russia [119]. In Europe, this taxon is currently treated under *Phiaris* [118].

***Phiarisbipunctana* (Fabricius, 1794)**

**Previous status:** Holarctic 

**Revised status:** No change

**Supported by DNA in BOLD:** Yes

**BINs:** BOLD:AAA6005

Sequences of *P. bipunctana* in BOLD are placed in the same BIN (BOLD:AAA6005) as sequences of *O. glaciana*, but form a separate distinct cluster. Specimens of *P. bipunctana* are from Canada (Manitoba, Yukon Territory) and Europe (Austria, Finland, Germany, Italy, Norway), and most share nearly identical DNA barcodes.

This species was first reported from North America by Landry et al. [32], with a specimen from Churchill, Manitoba collected in 2007. It has been suggested in the past that *glaciana* and *bipunctana* might be synonyms. Miller [120] examined both species and determined that they were not conspecific, and Landry et al. [32] provided a more detailed list of differences between the two taxa.

***Phiaris glaciana* (Möschler, 1860), comb. n.**

**Previous status:** Holarctic

**Revised status:** Nearctic only; transfer to *Phiaris*

**Supported by DNA in BOLD:** Yes

**BINs:** BOLD:AAA6005

*Argyroploce glaciana* is represented in BOLD by more than 300 specimens in a single BIN (BOLD:AAA6005), which is shared with specimens of *Phiaris bipunctana*. Although both species are very similar, previous studies [32,120] concluded that it is possible to differentiate them by wing pattern and genitalia, and the DNA data for both species form distinct and separate clusters. Specimens of *P. glaciana* in BOLD are from Alaska, Washington, and across much of Canada.

Lafontaine and Wood [122] listed *P. glaciana* as Holarctic, but it is possible they were referring to misidentified specimens of *P. bipunctana* instead, or even another species. We have not found any verified records of *P. glaciana* from Europe (including under other generic names), and this species is not included in the most recent European faunal list [118]. As such, *P. glaciana* is likely not Holarctic. Landry et al. [32] suggested that the similarity of *glaciana* to *bipunctana* meant that they should be placed in the same genus. We agree, based on both morphological and molecular data, and thus transfer *glaciana* to *Phiaris*.

***Phiaris siderana* (Treitschke, 1834)**

**Previous status:** Holarctic

**Revised status:** No change

**Supported by DNA in BOLD:** No (no specimens from North America in BOLD)

**BINs:** BOLD:AAJ2026

Only two specimens of *P. siderana* are present in BOLD: one from Norway and the other with no locality data. This species is widespread across Europe and parts of Asia, including Russia [119,123]. *Sericoris chalybeana* was described from California and is present in the Pacific Northwest and Montana [88]. This species is currently considered a junior synonym of *P. siderana*, and it has been treated as a subspecies in the past [71]. In Europe, this taxon is currently treated under *Celypha* [118].

***Selenodes concretana* (Wocke, 1862)**

**Previous status:** Holarctic

**Revised status:** No change

**Supported by DNA in BOLD:** Yes

**BINs:** BOLD:AAF4644

Six specimens of *S. concretana* are represented in BOLD (BIN BOLD:AAF4644): three labeled *Argyroploce concretana* (the current combination in Europe) from Finland, and three labeled *Selenodes concretana* from Canada (Northwest Territories and Yukon Territory); all appear to have identical DNA barcodes. Jalava and Miller [111] reported specimens from Eagle Summit, Alaska, and near Fairbanks, Alaska, which were the first North American records of this species.

***Celypha cespitana* (Hübner, [1817])**

**Previous status:** Holarctic (no status listed in Pohl et al. [28])

**Revised status:***Celypha cespitana* (Palearctic); North American cryptic sp. (Nearctic)

**Supported by DNA in BOLD:** Yes

**BINs:** BOLD:AAA9471

*Celypha cespitana* is represented in BOLD (BIN BOLD:AAA9471) by specimens from across Europe (Austria, Belgium, Bulgaria, Finland, France, Germany, Italy, Macedonia, Norway, United Kingdom). One specimen in the same BIN from China is identified as *C. flavipalpana* (possibly a misidentification). Several specimens identified as *C. cespitana* from North America are placed in BIN BOLD:AAA7669, although the majority of the nearly 200 specimens in that BIN are identified as *Olethreutes baccatana* (McDunnough, 1942). The two BINs (i.e., BOLD:AAA9471 and BOLD:AAA7669) are separated by 2.42% (p-dist).

Heinrich [25] synonymized the North American *Sericoris instrutana* Clemens, 1865 under the European *cespitana*, stating that there were no differences in [wing] pattern or genitalia between the two. DNA barcodes indicate that *C. cespitana* is not present in North America. It is not known if North American *C. cespitana* and *O. baccatana* in BIN BOLD:AAA7669 are indeed the same species or if barcodes are not sufficient to separate taxa in this group. If they are synonyms, *C. instrutana* would be the senior name, with *Sericoris poana* Zeller, 1875 being an additional junior synonym.

***Argyroploce aquilonana* Karvonen, 1932**

**Previous status:** Holarctic

**Revised status:** No change

**Supported by DNA in BOLD:** Yes

**BINs:** BOLD:AAB9941

There is a single BIN (BOLD:AAB9941) in BOLD representing *A. aquilonana*, with specimens from Canada (Manitoba, Northwest Territories, Nunavut, Yukon Territory), Greenland, and Finland. Jalava and Miller [111] reported this species (as *Olethreutes aquilonanus*) from the Ogilvie Mountains in the Yukon, which was the first North American record. They also synonymized this taxon with *Olethreutes kononenkoi*, extending the distribution of *A. aquilonana* into Russia. Nedoshivina [119] records this species from Chukotka in the Russian Far East.

***Argyroploce externa* (Eversmann, 1844)**

**Previous status:** Holarctic

**Revised status:** No change

**Supported by DNA in BOLD:** Yes

**BINs:** BOLD:AAI3239

Specimens of *A. externa* are placed in a single BIN (BOLD:AAI3239) with representatives from Europe (Finland, Norway) and Canada (Alberta, Northwest Territories, Yukon Territory). Jalava and Miller [111] reported the presence of the junior synonym “*Olethreutes dalecarlianus*” in North America, from the Ogilvie Mountains in the Yukon and Wellington, British Columbia. This name was synonymized under the Palearctic *Argyroploce externa* by Nedoshivina [124], making the senior taxon Holarctic.

***Hedya atropunctana* (Zetterstedt, 1840)**

**Previous status:** Holarctic

**Revised status:** No change

**Supported by DNA in BOLD:** Yes

**BINs:** BOLD:ABZ7645

In BOLD, sequences of *H. atropunctana* are placed in a single BIN (BOLD:ABZ7645) that includes specimens from Europe (Austria, Finland, Germany, Italy, The Netherlands, Norway, United Kingdom) and a single specimen from Alaska. Jalava and Miller [111] reported specimens of this species from the Ogilvie Mountains in the Yukon and the Dietrich River in Alaska, confirming that it is indeed Holarctic. Nedoshivina [119] lists many records of this species from Asia. Alipanah and Baixeras [125] reviewed the status of this genus and resurrected the combination *Hedya atropunctana* (which was previously placed in *Metendothenia*).

***Hedya ochroleucana* (Frölich, 1828)**

**Previous status:** Holarctic (no status listed in Pohl et al. [28])

**Revised status:***Hedya ochroleucana* (Palearctic); North American cryptic spp. (Nearctic)

**Supported by DNA in BOLD:** Yes

**BINs:** BOLD:AAC0586 (Europe); BOLD:ACE9334 (British Columbia); BOLD:AAC0585 (Canada, California)

Specimens of *H. ochroleucana* are placed in three BINs in BOLD: BOLD:AAC0586 contains specimens from Europe (Austria, Finland, Germany, The Netherlands, Norway), and the other two BINs (BOLD:ACE9334, BOLD:AAC0585) contain specimens from North America. The North American BINs are separated by 3.10% (p-dist), and both are separated from the European BIN by 7.35% (p-dist).

*Hedya ochroleucana* was described from Germany. DNA barcode data indicate that it is Palearctic, and that specimens identified as this species from North America represent different taxa. Three closely related taxa, currently considered synonyms, are described from North America: *nimbatana* Clemens, 1860, from Massachusetts; *contrariana* Walker, 1863, from Nova Scotia; and *consanguinana* Walsingham, 1879, from California. It is uncertain whether these North American names are indeed all synonyms, or to which of the North American BINs the names should be applied.

***Hedya nubiferana* (Haworth, [1811])**

**Previous status:** Introduced from Europe

**Revised status:** No change

**Supported by DNA in BOLD:** Yes

**BINs:** BOLD:AAA4552

A single BIN (BOLD:AAA4552) in BOLD contains nearly 100 specimens from the U.S., Canada, and Europe. The majority of specimens share a single identical barcode. This species was first reported from North America in 1913 from Nova Scotia and in 1914 from British Columbia [41]. It is currently distributed in the northeastern U.S. and Canada and the Pacific Northwest.

***Hedya salicella* (Linnaeus, 1758)**

**Previous status:** Introduced from Europe

**Revised status:** No change

**Supported by DNA in BOLD:** No (no specimens from North America in BOLD)

**BINs:** BOLD:AAD6484

BOLD contains a single BIN with sequences of *H. salicella* (BOLD:AAD6484), represented only by specimens from Europe (Austria, Finland, Germany, The Netherlands, Norway). Sabourin et al. [126] reported the first records of this species from North America, which include 1956 in Ontario, 1975 in Massachusetts, and 1985 in Missouri and Newfoundland.

***Ancylis comptana* (Frölich, 1828)**

**Previous status:** Holarctic

**Revised status:***Ancylis comptana* (Palearctic); North American cryptic sp. (Nearctic)

**Supported by DNA in BOLD:** Yes

**BINs:** BOLD:AAB2712 (North America); BOLD:AAB2713 (Austria, Finland, Italy, United Kingdom); BOLD:ABX5131 (Finland, Germany, Italy); BOLD:ADI5033 (Finland, Norway)

Specimens of *A. comptana* are placed in four main BINs in BOLD; there are other single specimens placed in other BINs that are likely misidentified. BOLD:AAB2712 contains specimens from the U.S. (Delaware, Florida, Illinois, Oklahoma, Texas) and Canada (Alberta, British Columbia, Manitoba, Quebec). The other three BINs contain specimens from Europe: BOLD:AAB2713 Austria, Finland, Italy, United Kingdom); BOLD:ABX5131 (Finland, Germany, Italy); and BOLD:ADI5033 (Finland, Norway).

*Ancylis comptana* was described from Germany. Although the European representatives of this species in BOLD fall into three separate BINs, these form a single cluster separate from the North American BIN. Thus, it appears that *A. comptana* is restricted to the Palearctic, and there is a separate unrecognized species in North America. Numerous junior synonyms of *A. comptana* from North America are currently associated incorrectly with that species, and it is likely that one of these would apply to the Nearctic populations (*conflexana* Walker, 1863 is the oldest). However, more extensive research is needed to determine the taxonomic status of this group in North America and to examine the multiple BINs in Europe. *Ancylis* are notoriously difficult to identify, and there are many serious taxonomic issues to resolve in this genus.

***Ancylis unguicella* (Linnaeus, 1758)**

**Previous status:** Holarctic

**Revised status:** No change

**Supported by DNA in BOLD:** Yes

**BINs:** BOLD:AAB3498

The BIN assignment listed here is from Gilligan et al. [33], who resolved the taxonomy for this group of *Ancylis*. They determined that *A. unguicella* is Holarctic, with a distribution that includes Western Europe east to Siberia and Japan in the Palearctic, and from Alaska and British Columbia east to Ontario and south to Colorado in the Nearctic [33].

***Ancylis uncella* ([Denis and Schiffermüller], 1775)**

**Previous status:** Holarctic (from Gilligan et al. [33])

**Revised status:** No change

**Supported by DNA in BOLD:** Yes

**BINs:** BOLD:AAA7191

The BIN assignment for *A. uncella* is from Gilligan et al. [33], who resolved the taxonomy for this group. *Ancylis uncella* was previously thought to be a Palearctic taxon, but Gilligan et al. [33] determined that it was conspecific with a North American species, *A. carbonana*, making it Holarctic. This species is distributed from Europe east to Siberia and Japan in the Palearctic and across southern Canada and the northeastern U.S. in the Nearctic [33].

***Eucosmomorpha albersana* (Hübner, [1813])**

**Previous status:** Palearctic (“Introduced from Europe” in Pohl et al. [28])

**Revised status:** Palearctic

**Supported by DNA in BOLD:** Yes

**BINs:** BOLD:AAF2360

Sequences of *E. albersana* in BOLD are placed in a single BIN (BOLD:AAF2360) representing Austria, Finland, Germany, The Netherlands, and Norway. This species was included by Pohl et al. [28] as introduced to North America. Although reported by various authors as such [127,128], Miller [129] subsequently concluded that North American specimens identified as this species were actually a new species that he described as *E. nearctica*. We are unaware of any valid records of the European *E. albersana* from North America.

***Enarmonia formosana* (Scopoli, 1763)**

**Previous status:** Introduced from Eurasia

**Revised status:** No change

**Supported by DNA in BOLD:** Yes

**BINs:** BOLD:AAC5227

*Enarmonia formosana* sequences are placed in a single BIN in BOLD (BOLD:AAC5227), which contains specimens from the U.S., Canada, and Europe. *Enarmonia formosana* is widely distributed from Western Europe and northern Africa to Asia Minor, Russia, and Siberia [123]. The first North American records are from British Columbia in 1989 [128]; it was subsequently found in western Washington in 1991 and in Oregon in 2000 [94]. The “cherry bark tortrix” is a common pest of numerous species of fruit trees [130].

***Rhyacionia buoliana* ([Denis and Schiffermüller], 1775)**

**Previous status:** Introduced from Europe

**Revised status:** No change

**Supported by DNA in BOLD:** Yes

**BINs:** BOLD:AAD1611 (Europe, Canada, U.S.); BOLD:ACE8290 (France and Spain)

Specimens of *R. buoliana* are placed in two BINs. BOLD:AAD1611 contains specimens from Europe (Austria, Finland, Germany, Italy, The Netherlands, Norway), Canada (Ontario), and the U.S. (Maryland). BOLD:ACE8290 contains specimens from France and Spain. These BINs form a single cluster and are less than 1% (p-dist) different, indicating that they represent a single species.

*Rhyacionia buoliana* was first recorded in North America in 1913 in New York [41]. It was reported from British Columbia in 1927, and although this initial population was eradicated, it was reported again from Vancouver in 1938 [41]. The “European pine shoot moth” is one of the most common pests of conifers. It is widely distributed and variable both in wing pattern and genitalia, resulting in a long list of synonyms described from Europe [27].

***Spilonota ocellana* ([Denis and Schiffermüller], 1775)**

**Previous status:** Introduced from Eurasia

**Revised status:** No change; possible cryptic species in Canada

**Supported by DNA in BOLD:** Yes

**BINs:** BOLD:ABZ4399 (Europe, North America); BOLD:AAA6641 (Canada)

Sequences of *S. ocellana* in BOLD are placed in two BINs: BOLD:ABZ4399, with specimens from Europe and North America, and BOLD:AAA6641, with specimens from Canada (British Columbia, Ontario, Quebec). It is not known if this second BIN represents a possible cryptic species or genetic variation.

*Spilonota ocellana* was introduced to the Nearctic likely before 1840, with the first report from Massachusetts in 1841 [41,131]. It became widely distributed in the northeastern U.S. and Canada, and was possibly introduced separately to the Pacific Coast, where it was common in Vancouver by 1912 [41,88]. This is a common orchard pest that is present in all apple-growing regions of the northern hemisphere [94].

***Spilonota laricana* (Heinemann, 1863)**

**Previous status:** Introduced from Europe

**Revised status:** No change; possible cryptic species in Europe

**Supported by DNA in BOLD:** Yes

**BINs:** BOLD:AAA7738 (Europe, Canada); BOLD:AAA7739 (Finland)

Sequences of *S. laricana* are placed in two BINs in BOLD: BOLD:AAA7738 contains specimens from Europe (Austria, France, Germany, Italy, The Netherlands, Norway, United Kingdom) and Canada (Newfoundland, New Brunswick, Nova Scotia, Ontario, Quebec), while BOLD:AAA7739 contains specimens from Finland. These BINs are separated by 4.1% (p-dist).

Historical records of this species in North America likely refer to *S. ocellana* [46]; thus it is difficult to determine when this species might have been introduced. It was reported from the northeastern U.S. by the Moth Photographers Group around 2010, and the DNA data in BOLD has confirmed that it is present and possibly widespread in eastern Canada. The second BIN (BOLD:AAA7739), with specimens from Finland, possibly represents a cryptic species in northern Europe.

***Eucosma cana* (Haworth, [1811])**

**Previous status:** Palearctic (not included in Pohl et al. [28])

**Revised status:** Holarctic or Introduced?

**Supported by DNA in BOLD:** Yes

**BINs:** BOLD:AAB4296

Specimens of *E. cana* in BOLD are placed in a single BIN (BOLD:AAB4296) with representatives from Europe (Austria, Bulgaria, Germany, Italy, Macedonia, The Netherlands, Norway, United Kingdom) and Canada, which includes records from Cape Breton Highlands National Park in Nova Scotia, Sable Island National Park in Nova Scotia, Forillon National Park in Quebec, and Gros Morne National Park in Newfoundland. Records from Canada date back to as early as 2009. This is the first report of *E. cana* in North America.

***Eucosma hohenwartiana* ([Denis and Schiffermüller], 1775)**

**Previous status:** Holarctic

**Revised status:** No change

**Supported by DNA in BOLD:** No (no specimens from North America in BOLD)

**BINs:** BOLD:AAB4295

Sequences of *E. hohenwartiana* in BOLD are placed in a single BIN (BOLD:AAB4295), with all of the specimens from Europe. Ferris et al. [102] recorded it from Northwest Alaska, adding it to the list of Holarctic tortricids. Wright and Gilligan [132] were unable to confirm this record, and there is no DNA evidence in BOLD to support or refute this conclusion.

***Pelochrista adamantana* (Guenée, 1845)**

**Previous status:** Holarctic

**Revised status:** Nearctic

**Supported by DNA in BOLD:** Yes

**BINs:** BOLD:AAF2211

The three specimens of *P. adamantana* with sequence data in BOLD are from the U.S., and are placed in a single BIN (BOLD:AAF2211). This species has been listed as potentially Holarctic in some checklists because of the statement in the original description that it was described from “ex Lapponia (?),” which could refer to Lapland in Finland. However, it has been excluded from European checklists since Rebel [133] determined that “it does not seem to be an inhabitant of this territory [the Palearctic].” Heinrich [24] appended “North America” to the location of the type specimen in an attempt to clarify the issue. Wright and Gilligan [134] were unable to locate the type, but they found no evidence of this species occurring in Europe.

***Pelochrista medullana* (Staudinger, 1879)**

**Previous status:** Introduced from Europe

**Revised status:** Not established in North America

**Supported by DNA in BOLD:** No (misidentifications and/or DNA barcodes do not separate species)

**BINs:** BOLD:AAE7175

In BOLD, two specimens labeled as *P. medullana* are placed in a single BIN (BOLD:AAE7175), along with specimens identified mostly as *P. caecimaculana* and a few other *Pelochrista* species from Europe. It is likely that the mixed species assignments to this BIN are the result of misidentifications. *Pelochrista medullana* was introduced into western North America for the biological control of *Centaurea* species (knapweed). This species was released in Idaho, Montana, Oregon, and British Columbia, but there is no evidence that it established in these locations [135,136].

***Epiblema simploniana* (Duponchel, 1835)**

**Previous status:** Holarctic

**Revised status:***Epiblema simploniana* (Palearctic); *Epiblema arctica* Miller, 1985, **stat. rev.** (Nearctic); other potential cryptic species

**Supported by DNA in BOLD:** Yes

**BINs:** BOLD:ACO4599 (*E. arctica*, Ogilvie Mtns., Canada); BOLD:ACO4600 (*E. arctica*, British Mtns., Canada); BOLD:AAF2136 (*E. simploniana*, Finland, Norway); BOLD:AAF2135 (*E. simploniana*, Finland); BOLD:ACJ7207 (*E. simploniana*, Austria, Italy)

Sequences of *E. simploniana* and its former junior synonym, *E. arctica*, are placed in five different BINs in BOLD: BOLD:ACO4599 contains specimens identified as *E. arctica* from the Ogilvie Mountains in the Yukon Territory, Canada; BOLD:ACO4600 contains specimens identified as *E. arctica* from the British Mountains in northwestern Yukon; BOLD:AAF2136 contains specimens identified as *E. simploniana* from Finland and Norway; BOLD:AAF2135 contains specimens labeled as *E. simploniana* from Finland; and BOLD:ACJ7207 contains specimens labeled as *E. simploniana* from Austria and Italy.

Miller [137] described *E. arctica* from specimens collected in Alaska. He later [138] determined that *E. arctica* was conspecific with *E. simploniana*, a Palearctic species recorded from the mountains of Central Europe, Scandinavia, western Russia, Siberia, and Mongolia [123]. DNA barcodes for this group segregate into several BINs that are separated geographically, with *E. arctica* in the Nearctic and *E. simploniana* in the Palearctic. In addition, we found what appear to be subtle but consistent morphological differences between *E. arctica* and *E. simploniana* that could be used to separate the two taxa (unpublished). Further research is necessary to determine why there are two separate BINs for *E. arctica* and three separate BINs for *E. simploniana*; this could indicate the possibility of one or more cryptic species in this group. Regardless, there is sufficient evidence to elevate *E. arctica* Miller, 1985, stat. rev., back to species status and restrict the distribution of *E. simploniana* to the Palearctic.

***Epiblema sticticana* (Fabricius, 1794)**

**Previous status:** Palearctic (not included in Pohl et al. [28])

**Revised status:** Holarctic or Introduced?

**Supported by DNA in BOLD:** Yes

**BINs:** BOLD:AAC0719

Specimens of this species are placed in a single BIN (BOLD:AAC0719) with representatives from Europe (Austria, Finland, Germany, Italy, The Netherlands, Norway, United Kingdom) and a single specimen from Canada. The specimen from Canada was collected in Gros Morne National Park in Newfoundland in 2009. It shares a 100% identical DNA barcode with 15+ specimens identified as *E. sticticana* from Europe. It appears that this taxon is either Holarctic or newly introduced to North America.

***Notocelia rosaecolana* (Doubleday, 1850)**

**Previous status:** Introduced from Europe

**Revised status:** No change

**Supported by DNA in BOLD:** Yes

**BINs:** BOLD:AAC1134 (Europe, North America)

Specimens of *N. rosaecolana* in BOLD are placed in a single BIN (BOLD:AAC1134) with representatives from Europe (Austria, Finland, Germany, The Netherlands, Norway, United Kingdom), Canada (Ontario, Quebec), and the U.S. (Illinois, Tennessee, Virginia, Washington). There is little genetic variation in this BIN, and many specimens share nearly identical DNA barcodes. A single specimen from the United Kingdom is placed in a separate BIN by itself (BOLD:AAC1135), but the DNA sequence is short and likely accounts for placement of this specimen.

There has been a long history of attempting to determine if the European *N. rosaecolana*, *N. trimaculana*, or both species were introduced to North America. The earliest record is from Smith [139], who reported *Eucosma suffusana* (a junior synonym of *N. trimaculana*) from New Jersey. Various other publications refer to *N. trimaculana* or *N. rosaecolana* as being the introduced taxon. Miller et al. [140] resolved the issue by discovering a morphological character that could be used to consistently separate the two taxa. They determined that *N. rosaecolana* is the only species of the two present in North America, and that prior literature records referring to *N. trimaculana* were likely incorrect.

***Notocelia cynosbatella* (Linnaeus, 1758)**

**Previous status:** Introduced from Europe

**Revised status:** No change; potential cryptic species and/or misidentifications

**Supported by DNA in BOLD:** Yes

**BINs:** BOLD:AAC3245 (Canada); BOLD:AAC9709 (Europe); BOLD:AAC3246 (Europe and Canada)

Sequences of *N. cynosbatella* are placed in three separate BINs in BOLD: BOLD:AAC3245 contains specimens from Canada (Alberta, British Columbia); BOLD:AAC9709 contains specimens from Europe (Austria, Norway, United Kingdom); and BOLD:AAC3246 contains specimens from Europe and Canada. The last BIN also contains some specimens labeled as *N. illotana*. BINs are separated by 2.4–3.4% (p-dist).

Mutuura [141] documented the first record of *N. cynosbatella* in North America from specimens collected near Vancouver, British Columbia in 1978. Pohl et al. [46] list the distribution as Alberta, British Columbia, Newfoundland, and Ontario. *Notocelia cynosbatella* is very similar to *N. illotana*, which is native to North America. Although Mutuura [141] provides a list of characters that can be used to separate the two species, the differences appear subtle, and genitalic dissection may be necessary for a positive identification. It is possible that the BIN containing only specimens from Canada could represent *N. illotana*, or that DNA barcodes in BOLD are not sufficient to separate these two species. It is also possible that there are cryptic species in this group.

***Gypsonoma parryana* (Curtis, 1835)**

**Previous status:** Holarctic

**Revised status:** No change

**Supported by DNA in BOLD:** No (misidentifications and/or DNA barcodes do not separate species)

**BINs:** BOLD:AAB1285

The BIN BOLD:AAB1285 contains specimens identified as several different species of *Gypsonoma*, including *G. parryana*, *G. nitidulana*, *G. fasciolana*, *G. salicicolana*, and *G. nebulosana*. The sequences cluster into several groups, but it is not clear if many of the specimens in this BIN are misidentified or if these DNA data are not sufficient to separate out species. Pohl et al. [46] list this species from Alaska, northwestern Canada, Manitoba, and Quebec. It is also recorded from Russia, in Novaya Zemlya and the Russian Far East [123].

***Gypsonoma nitidulana* (Lienig and Zeller, 1846)**

**Previous status:** Holarctic

**Revised status:** Palearctic

**Supported by DNA in BOLD:** No (misidentifications and/or DNA barcodes do not separate species)

**BINs:** BOLD:AAB1285

Sequences of this species are placed in BIN BOLD:AAB1285, along with sequences of the previous taxon and several other species (*G. parryana*, *G. nitidulana*, *G. fasciolana*, *G. salicicolana*, and *G. nebulosana*). It is not known if many of these are misidentifications or if DNA barcodes are not able to separate species in this group.

*Gypsonoma nitidulana* was reported from the Yukon by Lafontaine and Wood [122]; however, Pohl et al. [46] could not locate any voucher material, and declared the record to be erroneous. There is currently no evidence in the DNA data or collections to suggest *G. nitidulana* is present in North America.

***Gypsonoma aceriana* (Duponchel, 1842)**

**Previous status:** Introduced from Europe

**Revised status:** No change

**Supported by DNA in BOLD:** Yes

**BINs:** BOLD:AAB0379

Sequences of *G. aceriana* in BOLD are placed in a single BIN (BOLD:AAB0379) with specimens from Europe (Austria, Denmark, Germany, Italy, Norway, United Kingdom) and Canada. This species was first reported from North America in western Washington by Miller and LaGasa [142]. Several years later, Humble et al. [30] reported specimens of *G. aceriana* that were collected in southwestern British Columbia as early as 1980. Subsequent collecting by LaGasa and Humble et al. [30] confirmed that this species is established in the Pacific Northwest.

***Crocidosema plebejana* Zeller, 1847**

**Previous status:** Holarctic

**Revised status:***Crocidosema plebejana* (Palearctic); potentially several other species, none with a Holarctic distribution

**Supported by DNA in BOLD:** Yes

**BINs:** BOLD:AAA7083 (Australia); BOLD:ABY9664 (Africa); BOLD:ACE9795 (Europe, Egypt); BOLD:AAA7084 (California, Texas, Costa Rica)

Sequences of *C. plebejana* in BOLD are separated geographically into several BINs. BOLD:AAA7083 contains specimens only from Australia; BOLD:ABY9664 contains specimens only from Africa; BOLD:ACE9795 contains specimens from Europe and Egypt; and BOLD:AAA7084 contains specimens from California, Texas, and Costa Rica. There is seemingly no regional overlap in the BOLD data, suggesting that each of these BINs might be a separate taxon. Distance between BINs varies from 1.4% (p-dist) between BOLD:AAA7083 and BOLD:ACE9795 to 4.3% (p-dist) between BOLD:AAA7084 and the other three BINs.

*Crocidosema plebejana* was originally described from Europe, and an additional 13 names, described from various parts of the world, are currently considered synonyms, including several from the New World. Some other species described from Hawaii and South America are closely related [143,144]. Although *C. plebejana* is currently treated as a cosmopolitan species, it has been suggested that several of the synonyms are valid species [123]. Razowski et al. [145] resurrected from the list of synonyms *C. synneurota* Meyrick (described from the Galapagos) as a valid species and suggested other potential changes. The DNA data in BOLD suggest that at least populations on each continent might be separate taxa. Similarly, Lincango [146] in a multi-gene phylogenetic analysis found evidence of paraphyly in a complex of species around *C. plebejana*, and the specimens of *C. plebejana* from Europe did not group with those from South America. Moreover, there is extreme variation in both male and female genitalia. A more comprehensive molecular analysis is necessary to clarify the identity of the species involved. For now, we believe *C. plebejana* should be restricted to the Palearctic, and that none of the taxa involved in this complex are Holarctic in distribution.

***Crocidosema lantana* Busck, 1910**

**Previous status:** Holarctic?

**Revised status:** Neotropical; introduced elsewhere for biocontrol

**Supported by DNA in BOLD:** Yes (although no records for Florida)

**BINs:** BOLD:AAH5763 (Costa Rica, Jamaica, Australia, Madagascar, Kenya)

The sequences of *C. lantana* in BOLD are placed in a single BIN (BOLD:AAH5763), with specimens from Costa Rica, Jamaica, Australia, Madagascar, and Kenya.

This species was introduced to Hawaii from Mexico in 1902 to control *Lantana* [144]. It was later introduced to Australia from Hawaii around 1914, where it became common along the East Coast and prompted the description of several new names by Turner which were synonymized by Common [147]. *Crocidosema lantana* has been introduced to Kenya [148], and is also present in South Africa and apparently Madagascar based on DNA records. In North America, *C. lantana* is found in Florida.

***Rhopobota naevana* (Hübner, [1817])**

**Previous status:** Holarctic

**Revised status:** No change

**Supported by DNA in BOLD:** Yes

**BINs:** BOLD:AAA9812

In BOLD, the nearly 700 sequences representing this species fall into a single BIN (BOLD:AAA9812), with representatives from Canada, Europe, Japan, Pakistan, and the U.S. There is no evidence of any cryptic taxa.

This species is most likely Holarctic, being recorded from North America as early as 1863 and 1869 based on junior synonyms described from Hudson Bay, Canada and Massachusetts. Gillespie and Gillespie [41] state that it was probably introduced into western North America (Washington and Oregon) between 1912 and 1915.

***Epinotia trigonella* (Linnaeus, 1758)**

**Previous status:** Holarctic

**Revised status:** No change

**Supported by DNA in BOLD:** Yes

**BINs:** BOLD:AAB2504

Specimens identified as *E. trigonella* fall into a single BIN from Europe (Austria, Finland, France, Germany, Italy, Norway, United Kingdom) and Canada (Alberta, British Columbia, Manitoba, New Brunswick, Newfoundland, Northwest Territories). Sequences are clustered into three groups, with one consisting of mostly specimens identified as *E. indecorana* from Finland and Norway. Two specimens from Alaska identified as *E. trigonella* are placed in other BINs and most likely misidentified.

DNA data support a Holarctic distribution for this species. Pohl et al. [46] include the unpublished manuscript name *jasperana* Brown, 1980 [149], described from Jasper, Alberta, as a junior synonym of *E. trigonella*. Aarvik [118] treats *E. indecorana* and *E. trigonella* as separate species, and Karsholt et al. [48] follow this arrangement in their list of Lepidoptera from Greenland. DNA data in BOLD appear to support this separation, with sequences of *E. indecorana* clustering separately from those of *E. trigonella*.

***Epinotia solandriana* (Linnaeus, 1758)**

**Previous status:** Holarctic

**Revised status:** Holarctic?

**Supported by DNA in BOLD:** No (sequence clustering is ambiguous)

**BINs:** BOLD:AAA6716

Sequences of *E. solandriana* in BOLD are placed a single BIN (BOLD:AAA6716). However, sequences within this BIN are divided into two distinct clusters, with one representing North America (Alaska, California, Washington, Alberta, British Columbia, Manitoba, New Brunswick, Newfoundland, Quebec, Saskatchewan) and the other representing Europe (Finland, Germany, Italy, Norway). This clustering of sequences could suggest that the European and North American populations represent different species, but more study is needed to determine the genetic variability of this species.

*Epinotia solandriana* is currently considered to be Holarctic [28,149]. Martineau [150] suggested that this species was introduced from Europe to British Columbia in 1909. There is no other direct evidence of such; however, the clustering of sequences by continent in BOLD could also represent a genetic bottle-neck resulting from a potential introduction.

***Epinotia abbreviana* (Fabricius, 1794)**

**Previous status:** Introduced from Europe

**Revised status:** No change

**Supported by DNA in BOLD:** No (no specimens from North America in BOLD)

**BINs:** BOLD:AAE1784

*Epinotia abbreviana* is represented in BOLD with specimens from Europe placed in a single BIN (BOLD:AAE1784). This species was first recorded from North America by Dang [151], who documented specimens of *E. abbreviana* reared from St. John’s, Newfoundland in 1981 and 1988.

***Epinotia nisella* (Clerk, 1759)**

**Previous status:** Holarctic (no status listed in Pohl et al. [28])

**Revised status:** No change

**Supported by DNA in BOLD:** Yes

**BINs:** BOLD:ABZ1141 (Austria, Germany); BOLD:AAA7530 (Europe, Alaska); BOLD:AAA7528 (Canada, Alaska)

In BOLD, *E. nisella* is placed in three BINs: BOLD:ABZ1141 containing specimens from Austria and Germany; BOLD:AAA7530 containing specimens from Europe and Alaska; and BOLD:AAA7528 containing specimens rom Canada and Alaska. Mutanen et al. [31] provides details regarding DNA barcodes for this group.

Mutanen et al. [31] resolved the taxonomy of this and the next species (*E. cinereana*). *Epinotia nisella* has a long list of synonyms, owing to its variable forewing pattern. This species is currently distributed throughout Europe and Russia to China and Japan, and has even been recorded from Iceland [31]. In North America, it is found in the northern U.S. and across Canada north to the Yukon and Alaska.

***Epinotia cinereana* (Haworth, [1811])**

**Previous status:** Holarctic

**Revised status:** No change

**Supported by DNA in BOLD:** Yes

**BINs:** BOLD:AAA7532 (*E. cinereana*, Europe); BOLD:AAA7529 (*E. criddleana*, Canada); BOLD:ABX4876 (*E. cinereana*/*criddleana*, Europe, Canada)

The specimens of *E. cinereana* represented in BOLD are placed in three BINs: BOLD:AAA7532 containing specimens labeled as *E. cinereana* from Europe; BOLD:AAA7529 containing specimens labeled as *E. criddleana* from Canada; and BOLD:ABX4876 containing specimens labeled as *E. cinereana* from Europe and *E. criddleana* from Canada. Mutanen et al. [31] provide details regarding DNA barcodes for this group.

Mutanen et al. [31] resolved the taxonomy of this species, which was previously synonymized under *E. nisella*. They determined (based on morphology) that the North American *E. criddleana* is a junior synonym of *E. cinereana*, resulting in a Holarctic distribution. *Epinotia cinereana* is widely distributed across Europe, with scattered records through Russia to Japan and China [31]. In North America, it is found from Quebec and New Brunswick west to British Columbia and south to Iowa and Colorado [31].

***Epinotia columbia* (Kearfott, 1904)**

**Previous status:** Holarctic

**Revised status:** Nearctic; potential cryptic species in North America

**Supported by DNA in BOLD:** Yes

**BINs:** BOLD:AAF0407 (California, Canada); BOLD:AAE1754 (Canada)

*Epinotia columbia* is represented in BOLD with two BINs: BOLD:AAF0407 contains specimens from California and British Columbia, and BOLD:AAE1754 contains specimens from Canada (Alberta, Northwest Territories, Ontario, Quebec, Yukon Territory). Both are separated from the BIN containing *E. crenana* (former senior synonym) from Europe (BOLD:AAE1756). It is not known which BIN represents *E. columbia*, although it was described from British Columbia, and the historic distribution has been limited to western North America. Thus, it is likely that BOLD:AAE1754 represents an undescribed cryptic species.

Heinrich [24] synonymized *columbia* with *E. crenana* based on similarities in genitalia. In his unpublished Master’s thesis, R. L. Brown [149] elevated *E. columbia* back to species status; this change was formalized by J. Brown [26] in the tortricid world catalogue. Assuming many of the records from Canada represent a potential cryptic species (see above), the current distribution of *E. columbia* includes much of Canada west to British Columbia, Arizona, Nevada, Utah, and along the Pacific Coast to southern California [88]. There is no evidence that this species occurs in Europe, and records of such prior to 2005 are likely due to the previous synonymy with *E. crenana*.

***Epinotia nanana* (Treitschke, 1835)**

**Previous status:** Introduced from Europe

**Revised status:** No change

**Supported by DNA in BOLD:** Yes

**BINs:** BOLD:AAA8628 (Canada, Europe, U.S.); BOLD:ACF5528 (Europe)

Sequences of *E. nanana* fall into two BINs in BOLD. BOLD:AAA8628 contains specimens from Canada (New Brunswick, Ontario, Quebec, Prince Edward Island), the U.S. (Connecticut), and Europe (Finland, Norway). BOLD:ACF5528 contains specimens from other locations in Europe (Austria, Germany, Italy). Both BINs cluster together and are separated by only 1.09% (p-dist). As such, it is likely they represent a single taxon with some geographic structuring.

*Epinotia nanana* was originally described from Germany, and in the Palearctic, it is widely distributed across Europe, ranging into Siberia and Mongolia [123]. The first records in North America are based on species described by Kearfott that were later determined to be junior synonyms of *E. nanana* by Heinrich [24]. *Eucosma domonana* (Kearfott, 1907) was described from Framingham, Massachusetts, and *Epinotia piceafoliana* (Kearfott, 1908) was described from Essex County Park, New Jersey. The first record of *E. nanana* in western North America is from a single specimen collected in British Columbia at some point prior to 1965 [41]. This species is currently distributed from West Virginia and Ohio northeast to Prince Edward Island. It is not known if *E. nanana* established in British Columbia.

***Epinotia medioplagata* (Walsingham, 1895)**

**Previous status:** Holarctic

**Revised status:** No change

**Supported by DNA in BOLD:** No (no specimens from outside North America in BOLD)

**BINs:** BOLD:AAB6354

BOLD contains sequences of specimens from the U.S. and Canada placed in a single BIN (BOLD:AAB6354). There are no specimens from the Palearctic represented in BOLD. However, this species is also recorded from Magadan Region in the Russian Far East [152], and was considered to be Holarctic by Lafontaine and Wood [122].

***Epinotia cruciana* (Linnaeus, 1761)**

**Previous status:** Holarctic

**Revised status:** No change; potential cryptic species

**Supported by DNA in BOLD:** Yes

**BINs:** BOLD:ABX2894 (Canada); BOLD:ACE9994 (Canada, Italy); BOLD:AAC2644 (Canada, Europe)

Sequences of *E. cruciana* fall into three BINs: BOLD:ABX2894 contains specimens from Canada (Alberta, British Columbia, Northwest Territories); BOLD:ACE9994 contains specimens from Canada (Alberta, British Columbia, Manitoba, Northwest Territories) and two specimens from Italy; and BOLD:AAC2644 contains specimens from Europe (Austria, Finland, Germany, The Netherlands, Norway, Russia, United Kingdom) and much of Canada. The first BIN containing specimens only from Canada is more distant (2.2% p-dist) from the other two BINs, which are only separated by 1.2% (p-dist). It is likely that one or both of the BINs containing specimens from Europe represent the true *E. cruciana*.

This species was described from Sweden and has at least 15 junior synonyms, several of which are from North America [27]. *Epinotia cruciana* ssp. *alaskae* was described from Alaska; *cockleana* was described from western Canada; *direptana* was described from Hudson Bay, Canada; *E. cruciana* ssp. *lepida* was described from New Hampshire; and *vilisana* was described from Hudson Bay, Canada. 

***Epinotia seorsa* Heinrich, 1924**

**Previous status:** Holarctic?

**Revised status:** Nearctic

**Supported by DNA in BOLD:** No (this species not present in BOLD)

**BINs:** N/A

The known distribution of *E. seorsa* includes western Canada (Alberta, British Columbia) and California [153]. There are no records of this species from Europe and no sequences in BOLD. Pohl et al. [28] included the unpublished manuscript name *blanchardi* [149] as a junior synonym, which extends the range of this species into Texas. However, we suspect this is an error because specimens of the two taxa in the USNM do not appear to be similar.

***Epinotia salicicolana* Kuznetsov, 1968**

**Previous status:** Introduced?

**Revised status:** Holarctic or Introduced?

**Supported by DNA in BOLD:** No (no specimens from North America in BOLD)

**BINs:** N/A

*Epinotia salicicolana* was described from the Kuril Islands in the Russian Far East and has been also recorded from Korea, Japan, China, and Taiwan [154,155]. It was first reported from North America in 2007 from British Columbia (E-Fauna BC: Electronic Atlas of the Fauna of British Columbia, www.efauna.bc.ca). Pohl et al. [46] listed this species from British Columbia, presumably based on the 2007 record. Only one specimen with a barcode is present in BOLD, and it is from Japan (and not assigned to a BIN). Although there are no records prior to 2007 from North America, this species could be Holarctic or introduced.

***Dichrorampha vancouverana* McDunnough, 1935**

**Previous status:** Holarctic

**Revised status:** No change

**Supported by DNA in BOLD:** Yes

**BINs:** BOLD:AAC1223

Sequences of *D. vancouverana* are placed in a single BIN, BOLD:AAC1223, with specimens from Europe (Austria, Finland, Germany, Italy, Norway, United Kingdom) and Canada (Alberta, British Columbia). As the name suggests, *D. vancouverana* was described from Vancouver Island, British Columbia. Miller [156] recognized that *D. vancouverana* was the same species as the European *D. gueneeana* (Obraztsov, 1953) and synonymized the two names.

The taxonomic history of this species is somewhat confusing. Denis and Schiffermüller [157] described *Tortrix politana* from Austria (now a junior synonym of *Lathronympha strigana*). Guenee [158] referred to an undescribed species of *Dichrorampha* (now *vancouverana*) in Europe as *politana*; however, this was clearly a misidentification. Obraztsov [159] assigned the name *gueneeana* as a replacement name for Guenee’s misidentified *politana*. Because Guenee’s reference to *politana* was simply a misidentification, it is likely that Obraztsov’s *gueneeana* is invalid, because this was assigned as a replacement name and was not a new species description. Regardless, the species previously referred to as *D. gueneeana* in Europe can now be correctly referred to as the Holarctic *D. vancouverana*.

***Dichrorampha sedatana* (Busck, 1906)**

**Previous status:** Holarctic

**Revised status:** Holarctic?

**Supported by DNA in BOLD:** No (misidentifications and/or DNA barcodes do not separate species)

**BINs:** BOLD:AAA8637 (D. sedatana, D. aeratana); BOLD:AAA8636 (D. sedatana, D. acuminatana)

*Dichrorampha sedatana* can be problematic to separate from similar species such as *D. aeratana*, *D. acuminatana*, *D. plumbana*, and *D. dana* based only on wing pattern. A genitalic dissection is necessary to confirm identity in this group, and even then, identification is difficult due to variation. The majority of *Dichrorampha* in BOLD are not dissected, and thus, species identifications are sometimes questionable, especially when multiple species are assigned to the same BIN. Sequences of *D. sedatana* in BOLD are placed in two BINs. BOLD:AAA8636 contains specimens labeled as *D. sedatana* from Canada and specimens labeled as *D. acuminatana* from Europe. BOLD:AAA8637 contains specimens labeled as *D. sedatana* from Europe, specimens labeled as *D. aeratana* from Europe and Canada, and specimens labeled as *D. tarmanni* from Italy. Relatively low genetic diversity in both BINs suggests that multiple names in each BIN are a result of misidentifications, or that multiple species within this group share nearly identical DNA barcodes. It is not possible to determine the status of these taxa without extensive examination of the specimens from this group represented in BOLD.

*Dichrorampha sedatana* is currently treated as Holarctic [123]. It has been noted in the past that *D. sedatana* specimens identified in Europe seem to be different from those identified in North America (K. Larsen, pers. comm.). However, the BOLD data are currently not sufficient to support this conclusion because of apparent issues with species identifications. Pohl et al. [46] noted that the use of *D. plumbana* (Scopoli, 1763) for North American populations is erroneous.

***Dichrorampha odorata* Brown and Zachariades, 2007**

**Previous status:** Introduced?

**Revised status:** Probably native to southern Florida/Caribbean

**Supported by DNA in BOLD:** No (this species not present in BOLD)

**BINs:** N/A

This species was described from Jamaica in 2007, but was found in Florida in 2014 feeding on weeds. There is no evidence it is introduced, and thus, it is probably native to the region and simply not detected in Florida until recently. This species is not represented in BOLD.

***Dichrorampha petiverella* (Linnaeus, 1758)**

**Previous status:** Introduced from Europe

**Revised status:** No change

**Supported by DNA in BOLD:** Yes

**BINs:** BOLD:AAC9827

Specimens of *D. petiverella* in BOLD are placed in a single BIN (BOLD:AAC9827) representing Europe (Austria, Finland, Germany, Italy, Norway, United Kingdom) and North America (Ontario, Quebec, Washington).

Roberts [160] documented this species in North America for the first time when he collected specimens from Washington County, Maine in 1989. BOLD records indicate that *D. petiverella* is also present in Ontario, Quebec, and Washington. Pohl et al. [46] recorded this species from Newfoundland, Nova Scotia, and Prince Edward Island.

***Dichrorampha acuminatana* (Lienig and Zeller, 1846)**

**Previous status:** Introduced from Europe

**Revised status:** No change

**Supported by DNA in BOLD:** No (misidentifications and/or DNA barcodes do not separate species)

**BINs:** BOLD:AAA8637 (D. sedatana, D. aeratana); BOLD:AAA8636 (D. sedatana, D. acuminatana)

See *D. sedatana* for a discussion of the DNA data for this group of species. *Dichrorampha acuminatana* was first discovered in North America in 2001 in Washington County, Maine [161]. This species has also been recorded from Vermont, with the first collection in 2003 [162], and there are photo records from Ontario. Pohl et al. [46] recorded this species from New Brunswick.

***Dichrorampha montanana* (Duponchel, 1843)**

**Previous status:** Introduced from Europe

**Revised status:** No change; cryptic species in Europe

**Supported by DNA in BOLD:** Yes

**BINs:** BOLD:AAE0714 (Europe, Canada; *D. montanana*); BOLD:AAE0715 (Europe; cryptic sp.)

*Dichrorampha montanana* is represented in BOLD in two BINs: BOLD:AAE0715 contains specimens from Europe (Austria, France, Germany, Italy, Switzerland), and BOLD:AAE0714 contains specimens from Europe (Austria, Italy, Macedonia, Switzerland, United Kingdom) and Canada (Newfoundland). It is assumed that BOLD:AAE0714 is the “true” *D. montanana* because it contains a specimen from Canada that was verified via dissection. Huemer [163] stated that morphological and genetic studies indicate a complex of two species in this group, and we assume that BOLD:AAE0715 does indeed represent a separate taxon. Pohl et al. [46] recorded *D. montanana* for the first time from North America with specimens from Newfoundland that were confirmed via genitalic dissection.

***Dichrorampha aeratana* (Pierce and Metcalfe, 1915)**

**Previous status:** Palearctic (not included in Pohl et al. [28])

**Revised status:** Introduced from Europe

**Supported by DNA in BOLD:** No (misidentifications and/or DNA barcodes do not separate species)

**BINs:** BOLD:AAA8637 (D. sedatana, D. aeratana); BOLD:AAA8636 (D. sedatana, D. acuminatana)

See *D. sedatana* for a discussion of the DNA data for this group of species. Sabourin [162] reported *D. aeratana* from North America with specimens first collected in 1992 in Vermont. Sabourin [162] also reported a specimen from Quebec collected in 1994, and there are photo records of this species from Ontario.

***Grapholita molesta* (Busck, 1916)**

**Previous status:** Introduced from Asia

**Revised status:** No change

**Supported by DNA in BOLD:** Yes

**BINs:** BOLD:AAB0523

In BOLD, specimens of *G. molesta* fall into a single BIN (BOLD:AAB0523) with a worldwide distribution. The earliest records of *G. molesta* in North America are from Washington, D.C., where it was apparently introduced in flowering cherry from Japan in 1912 or 1913 [164]. It has since spread throughout most of the U.S. and southern Canada. The “oriental fruit moth” is an important cosmopolitan pest of stone-fruit crops.

***Grapholita delineana* Walker, 1863**

**Previous status:** Introduced from Asia

**Revised status:** No change

**Supported by DNA in BOLD:** No (no specimens from North America in BOLD)

**BINs:** BOLD:AAY2279 (Pakistan, Japan); BOLD:ABW6517 (South Korea)

Specimens of *G. delineana* in BOLD are placed in two BINs: BOLD:AAY2279 contains specimens from Pakistan and Japan, and BOLD:ABW6517 contains a single specimen from South Korea. It is possible the South Korean specimen is misidentified.

*Grapholita delineana* is a native of East Asia that spread into Europe, Asia Minor, Transcausasia, and North America with the commercial production of hemp [123,165]. The earliest records from North America are from Wisconsin and Kentucky in 1943, as reported by Miller [165]. It is currently widely distributed in the eastern U.S., and there are records from Ontario [46,165]. Cranshaw et al. [166] found *G. delineana* to be present in hemp fields in eastern Colorado in 2017–2018, and it is likely that this species will continue to spread with expanded industrial hemp production.

***Grapholita aureolana* (Tengström, 1848)**

**Previous status:** Holarctic

**Revised status:** No change

**Supported by DNA in BOLD:** Yes

**BINs:** BOLD:AAE7252

Sequences of *Grapholita aureolana* fall into a single BIN in BOLD (BOLD:AAE7252), with specimens from Europe (Austria, Finland) and Canada. Pohl et al. [46] were the first to report this species from North America based on specimens in BOLD that were confirmed with genitalic dissections.

***Cydia coniferana* (Saxesen, 1840)**

**Previous status:** Introduced from Europe

**Revised status:** No change; potential cryptic species in western North America

**Supported by DNA in BOLD:** Yes

**BINs:** BOLD:AAC6167 (Europe and Canada); BOLD:AAC6168 (western U.S.)

*Cydia coniferana* is represented in BOLD by sequences that fall into two BINs: BOLD:AAC6167 contains specimens from Europe (Austria, Finland, Germany, Norway, United Kingdom) and Canada (Ontario), and BOLD:AAC6168 contains specimens from the western U.S. (Washington, New Mexico).

This species was first reported from North America by Schaffner [167] for several adults reared from the bark of red pine in New York. It was assumed that this population did not establish. LaGasa and Passoa [168] reported that this same species was also collected in Thurston County, Washington in 2000. Additional surveys in 2005 found specimens throughout most of western Washington, from the border of British Columbia to Oregon.

Based on the DNA sequence data, it seems that a population of *C. coniferana* did establish in eastern North America, with one specimen collected in 2015 from Peterborough in Central Ontario represented in BOLD. It is likely that the populations in the Pacific Northwest and elsewhere in western North America (New Mexico) represent a cryptic species. There are also records of this species from the Russian Far East [123]; these and other records from the region (e.g., Siberia) should be checked carefully to ensure they are indeed *C. coniferana*. 

***Cydia nigricana* (Fabricius, 1794)**

**Previous status:** Introduced from Europe

**Revised status:** No change; potential cryptic species in North America

**Supported by DNA in BOLD:** Yes

**BINs:** BOLD:AAA7614 (Europe and Canada); BOLD:AAE7682 (North America)

Sequences of *C. nigricana* in BOLD are placed into two separate BINs: BOLD:AAA7614 contains specimens from Europe (Austria, Finland, Germany, The Netherlands, Norway, United Kingdom) and Canada (Alberta, Manitoba, Ontario, Saskatchewan), and this BIN likely represents the true *C. nigricana*, which was described from Great Britain; the second BIN (BOLD:AAE7682) contains specimens from only North America (Oklahoma, Ontario, Quebec), and represents either a group of misidentified specimens or a potential cryptic species. There are two junior synonyms of *C. nigricana* that were described from the U.S. (*dandana* Kearfott, 1907 and *novimundi* Heinrich, 1920), and it is possible that one or both of these names could be applied to specimens in BOLD:AAE7682. *Cydia nigricana* was introduced from Europe, and first found in North America in eastern Canada in 1893 and in British Columbia in 1933 [46].

***Cydia pomonella* (Linnaeus, 1758)**

**Previous status:** Introduced from Europe

**Revised status:** No change

**Supported by DNA in BOLD:** Yes

**BINs:** BOLD:AAA3532

Sequences of *C. pomonella* in BOLD are placed into a single BIN (BOLD:AAA3532) that consists of specimens collected throughout the world. There is no evidence of any cryptic species. The earliest record of *C. pomonella* in North America is a report of wormy apples and pears in the vicinity of Boston in 1819 [164]. The “codling moth” is one of the most widespread and important pest of apple, pear, and walnuts in the world.

***Cydia saltitans* (Westwood, 1858), stat. rev.**

**Previous status:** Introduced

**Revised status:** Neotropical; not present in the U.S.

**Supported by DNA in BOLD:** Yes

**BINs:** BOLD:AAA0992

The name for the “Mexican jumping bean” moth has traditionally been cited as *Cydia deshaisiana* Lucas, 1858 in most modern publications. Although this name was published by Lucas in November, 1858 [169], his account (in French) is not accompanied by anything that can be construed as a description. As such, the name *deshaisiana* is a *nomen nudum*. The behavior of this insect (“jumping seeds”) had actually been described a year earlier by Westwood in 1857 [170], although he did not provide a formal description or assign a name in that paper. On June 7, 1858, at the meeting of the London Entomological Society, Westwood read a paper describing the same species (in Latin) under the name *Carpocapsa saltitans* [171,172].

Determining the exact date this description was published is difficult. The Proceedings of the Entomological Society of London were published on an irregular basis, most often quarterly. Wheeler [173] listed dates of publication by volume, part, and page number. It is possible that the June, 1858 proceedings would have been published in the next volume, which was delivered to the Society on July 5, 1858. However, because the exact page number is unknown, it is also not known if Westwood’s description was included in the July volume or if there was a delay in the proceedings appearing in print. We do know that Westwood’s account appeared in print on page 27 of the summary volume, Proceedings of the Entomological Society of London 1858–1859 [172], which appears to have been published as part of The Transactions of the Entomological Society of London, New Series, Volume V, 1858–1861, because the index that lists Westwood’s account includes the years 1858 through 1861. However, the 1858 issue of The Zoologist [171] also included a transcription of Westwood’s account. The exact publication date of Volume 16 of The Zoologist is not known; however, none of the dates in the volume are past 1858, and thus, it is assumed that it was printed in late 1858 or early 1859. As such, The Zoologist [171] is the earliest publication for which we can confirm a date for Westwood’s description of *C. saltitans*, unless we simply use the June 7, 1858 date when the paper was first read and assume it was published in the next issue of the Proceedings in July. Regardless, the correct name for the “Mexican jumping bean” moth is *Cydia saltitans* (Westwood, 1858), stat. rev.

The only specimens in BOLD are from Costa Rica in (BOLD:AAA0992), and there is no evidence of this species occurring north of Mexico. “Mexican jumping beans” are routinely imported into the U.S. and adult moths eventually emerge from the seeds. However, there are no records of this species being found in the wild in the U.S., and the distribution of *C. saltitans* appears to be restricted the northern states of Sinoloa and Sonora in Mexico.

***Cydia cornucopiae* (Tengström, 1869)**

**Previous status:** Holarctic?

**Revised status:** No change

**Supported by DNA in BOLD:** No (no specimens from North America in BOLD)

**BINs:** BOLD:AAF7704

This species was first reported from North America in Alaska by Ferris et al. [102]. It is otherwise known from Finland to the Russian Far East [123]. The only specimens in BOLD are from Finland and are placed in a single BIN (BOLD:AAF7704).

***Thaumatotibia leucotreta* (Meyrick, 1913)**

**Previous status:** Introduced

**Revised status:** Not established in North America

**Supported by DNA in BOLD:** Yes

**BINs:** BOLD:AAE7729

This species is native to Africa. A single male specimen was found in a pheromone trap in Ventura County, California in July, 2008 [174]. Although this find prompted extensive additional surveys, no other individuals were found. As such, this was assumed to be an isolated incident and this species was never established in California. There is a single specimen in BOLD (sample ID CCDB-10308-H02) listed as collected in New Jersey; however, this is an intercepted larva that did not originate from North America.

***Coniostola isabelae* Razowski and Landry, 2008**

**Previous status:** Neotropical (no status listed in Pohl et al. [28])

**Revised status:** No change

**Supported by DNA in BOLD:** No (this species not present in BOLD)

**BINs:** N/A

This species was described from the Galapagos Islands. Razowski and Becker [175] reported “several specimens” from San Benito, Texas, along with specimens from the Virgin Islands (St. Thomas) and British Virgin Islands (Guana Island). This species is not represented in BOLD. Because there is no evidence of introduction, it is likely this species is Neotropical and simply not discovered in southern Texas until recently.

**Subfamily CHLIDANOTINAE**

***Auratonota dispersa* Brown, 1990**

**Previous status:** Exotic, not established (extralimital in Pohl et al. [28])

**Revised status:** No change

**Supported by DNA in BOLD:** No (insufficient records in BOLD)

**BINs:** BOLD:AAJ9640

This species is native to Central America, e.g., Guatemala to Panama [176]. It has been reported from the North American fauna because a single male was collected in a blacklight trap at an inspection station in Miami, but there is no record that it is established in Florida [176]. A single specimen from Costa Rica is represented in BOLD.

## 4. Conclusions

Determining whether a species is natively Holarctic or a relatively recent immigrant to North America from Eurasia is often difficult, even with the assistance of DNA data. Based on an established set of criteria, we evaluated 151 species of Tortricidae putatively documented from North America and hypothesize their origin. A summary of the data is presented in Table 1. The most significant finding is the difference in the number of taxa previously assumed to be Holarctic and the number that we determined to be Holarctic: 76 species were previously assumed to be Holarctic, whereas DNA and other data indicate that a maximum of 58 of these are actually Holarctic (separating Holarctic from introduced is not possible in some cases; thus, the actual number is likely less than 58). Hence, prior assumptions regarding the Holarctic distribution of Tortricidae may be overestimated by more than 20%, which is not surprising, given other recent studies, e.g., those of Landry et al. [32] and Gilligan et al. [33]. The primary reason for this discrepancy appears to be the presence of cryptic species in the Nearctic that were incorrectly identified as Palearctic taxa by early taxonomists.

The overall number of tortricids introduced to North America is similar to those recently reported by other authors, e.g., Pohl et al. [46]. A total of 46 species are determined to be introduced (with another 5–6 species that may be introduced or Holarctic), and the majority of these are well-documented pest species. Given that the total number of Tortricidae in North America is approximately 1400 (estimated from the new North American checklist, in preparation), introduced taxa account for around 3% of the total number of species. This percentage is higher than most other families, exceeded only by Gelechiidae (~4%) and Pyralidae (~6%) [46]. In addition, the overall number of introduced Tortricidae in North America in relation to other families is much higher than would be expected. Sailer [177] and Wheeler and Hoebeke [178] estimated that approximately 150–200 species of introduced Lepidoptera species are present in North America. Tortricidae comprise approximately 10% of the North American Lepidoptera fauna, so it might be expected that a corresponding percentage of introduced species would be tortricids. Instead, it appears that somewhere between 23–30% of the total number of Lepidoptera introduced to North America are Tortricidae. Since 1900, the rate of introduction of exotic Tortricidae has been relatively constant, with approximately one new introduced species discovered every three years.

The overall importance of introduced taxa cannot be overstated, and the environmental and economic costs from invasive species continue to increase. Hence, it is critically important to recognize these pests and distinguish them from species that are natively Holarctic. Based on the entire order Lepidoptera, the superfamily Tortricoidea has the third highest number of economically important species (i.e., 687 species worldwide), trailing only Noctuoidea, with 1034 pests, and Pyraloidea, with 748 pests [179]. Furthermore, the global diversity of tortricids is considerably less than that of the latter two groups, suggesting that members of this superfamily have a higher potential to inflict economic damage. Among microlepidoptera, tortricid larvae vastly dominate interception numbers at U.S. ports of entry. The relevance of these interceptions and their potential subsequent introductions to American agriculture appear to be increasing, with economically important species like the European grapevine moth and light brown apple moth triggering significant quarantine actions, both domestically and abroad. As such, it is important to continue surveying for immigrant Tortricidae and to continue research on these taxa to determine whether species in question are introduced or Holarctic.

## Figures and Tables

**Table 1:** Summary of immigrant Tortricidae and their previous and current status. Changes of status are noted in bold (Afro = Afrotropical; Aust = Australia; Eur = Europe; H = Holarctic; I = Introduced; N = Nearctic; Neo = Neotropical; P = Palearctic).

Taxon	Previous Status	Revised Status	Notes
**Subfamily TORTRICINAE**			
*Acleris forsskaleana* (Linnaeus, 1758)	I-Eur	I-Eur	
*Acleris holmiana* (Linnaeus, 1758)	I-Eur	I-Eur	
*Acleris comariana* (Zeller, 1846)	I-Eur	I-Eur	
*Acleris rhombana* ([Denis and Schiffermüller], 1775)	H?	**I-Eur**	
*Acleris notana* (Donovan, [1806])	I-Eur	**I?**	
*Acleris implexana* (Walker, 1863)	H	**N**	
*Acleris ferrumixtana* (Benander, 1934), stat. rev.	syn. *implexana*	**H**	Revised status
*Acleris schalleriana* (Linnaeus, 1761)	H	**P**	
*Acleris viburnana* (Clemens, 1860), stat. rev.	syn. *schalleriana*	**N**	Revised status
*Acleris variegana* ([Denis and Schiffermüller], 1775)	I-Eur	I-Eur	
*Acleris hastiana* (Linnaeus, 1758)	H	**P**	
*Acleris pulverosana* (Walker, 1863), stat. rev.	syn. *hastiana*	**N**	Revised status
*Acleris arcticana* (Guenée, 1845)	H	H	
*Acleris robinsoniana* (Forbes, 1923)	H?	**N**	
*Acleris logiana* (Clerck, 1759)	H	**P**	
*Acleris placidana* (Robinson, 1869), stat. rev.	syn. *logiana*	**N**	Revised status
*Acleris maccana* (Treitschke, 1835)	H	H	
*Acleris lipsiana* ([Denis and Schiffermüller], 1775)	H?	**P**	
*Acleris capizziana* Obraztsov, 1963	N	N	
*Acleris scabrana* ([Denis and Schiffermüller], 1775)	H	H	
*Acleris effractana* (Hübner, 1822)	H	H	
*Acleris emargana* (Fabricius, 1775)	P	P	
*Cnephasia longana* (Haworth, [1811])	I-Eur	I-Eur	
*Cnephasia asseclana* ([Denis and Schiffermüller], 1775)	I-Eur	I-Eur	
*Cnephasia stephensiana* (Doubleday, 1849)	I-Eur	I-Eur	
*Eana argentana* (Clerck, 1759)	H	**P**	
*Eana osseana* (Scopoli, 1763)	H	**P**	
*Aethes deutschiana* (Zetterstedt, 1840)	H	**P**	
*Aethes rutilana* (Hübner, 1818)	H?	**H**	
*Aethes smeathmanniana* (Fabricius, 1781)	H	H	
*Agapeta zoegana* (Linnaeus, 1767)	I-Eur	I-Eur	
*Cochylidia subroseana* (Haworth, [1811])	H	H	
*Cochylis atricapitana* (Stephens, 1852)	I-Eur	I-Eur	
*Neocochylis dubitana* (Hübner, 1799)	H	H	
*Eugnosta argyroplaca* (Meyrick, 1931)	N	**P**	
*Eugnosta californica* Razowski, 1986	N	N	Not present in the U.S.
*Eugnosta chemsakiana* (Razowski, 1986)	N	N	Not present in the U.S.
*Phtheochroa vulneratana* (Zetterstedt, 1839)	H	**P**	
*Rolandylis maiana* (Kearfott, 1907)	H?	**N**	
*Spinipogon harmozones* Razowski, 1986	Neo	Neo	First record in the U.S.
*Thyraylia nana* (Haworth, [1811])	H	**H or I?**	
*Eulia ministrana* (Linnaeus, 1758)	H	H	
*Pandemis cerasana* (Hübner, 1786)	I-Eur	I-Eur	
*Pandemis heparana* ([Denis and Schiffermüller], 1775)	I-Eur	I-Eur	
*Argyrotaenia franciscana* (Walsingham, 1879)	N	N	Expanded range
*Choristoneura albaniana* (Walker, 1863)	H	H	
*Archips oporana* (Linnaeus, 1758)	I-Eur	I-Eur	
*Archips xylosteana* (Linnaeus, 1758)	I-Eur	I-Eur	
*Archips rosana* (Linnaeus, 1758)	I-Eur	I-Eur	
*Archips podana* (Scopoli, 1763)	I-Eur	I-Eur	
*Archips fuscocupreanus* Walsingham, 1900	I-Eur	I-Eur	
*Cacoecimorpha pronubana* (Hübner, 1822)	I-Eur	I-Eur	
*Dichelia histrionana* (Frölich, 1828)	I-Eur	I-Eur	
*Clepsis spectrana* (Treitschke, 1830)	I-Eur	I-Eur	
*Clepsis danilevskyi* Kostiuk, 1973	H	H	
*Clepsis consimilana* (Hübner, 1822)	I-Eur	I-Eur	
*Clepsis moeschleriana* (Wocke, 1862)	H	H	
*Clepsis illustrana* (Krogerus, 1936)	H	**H?**	
*Epiphyas postvittana* (Walker, 1863)	I-Aust	I-Aust	
*Ditula angustiorana* (Haworth, [1811])	I-Eur	I-Eur	
*Sparganothis rubicundana* (Herrich-Schäffer, 1856)	H?	**H**	
*Sparganothis praecana* (Kennel, 1900)	H	H	
*Platynota stultana* Walsingham, 1884	N	N	Introduced to Europe
**Subfamily OLETHREUTINAE**			
*Endothenia quadrimaculana* (Haworth, [1811])	I-Eur	I-Eur	
*Endothenia gentianaeana* (Hübner, [1799])	I-Eur	**P**	Previously misidentified
*Tia enervana* (Erschoff, 1877)	H	H	
*Bactra lancealana* (Hübner, [1799])	H	H	
*Bactra furfurana* (Haworth, [1811])	H	H	
*Bactra priapeia* Heinrich, 1923	Neo	Neo	Described from U.S.
*Lobesiodes euphorbiana* (Freyer, 1842)	I-Eur	I-Eur	
*Lobesia bicinctana* (Duponchel, 1843)	H	**P**	
*Lobesia spiraeae* (McDunnough, 1938), stat. rev.	syn. *bicinctana*	**N**	Revised status
*Lobesia botrana* ([Denis and Schiffermüller], 1775)	I	**P**	Eradicated from U.S.
*Apotomis capreana* (Hübner, [1817])	H	H	
*Apotomis infida* (Heinrich, 1926)	H	H	
*Cymolomia hartigiana* (Ratzeburg, 1840)	P	**I-Eur**	
*Orthotaenia undulana* ([Denis and Schiffermüller], 1775)	H	H	
*Syricoris lacunana* ([Denis and Schiffermüller], 1775)	P	**H**	
*Olethreutes metallicana* (Hübner, 1796)	H	**H?**	
*Olethreutes nordeggana* (McDunnough, 1922)	H	H	
*Olethreutes heinrichana* (McDunnough, 1927)	H	H	
*Olethreutes schulziana* (Fabricius, 1777)	H	H	
*Olethreutes turfosana* (Herrich-Schäffer, 1851)	H	H	
*Olethreutes septentrionana* (Curtis, 1835)	H	H	
*Olethreutes inquietana* (Walker, 1863)	H	H	
*Olethreutes mengelana* (Fernald, 1894)	N	**H**	
*Olethreutes exaridanus* Kuznetsov, 1991	H	H	
*Olethreutes palustrana* (Lienig and Zeller, 1846)	H	H	
*Phiaris bipunctana* (Fabricius, 1794)	H	H	
*Phiaris glaciana* (Möschler, 1860), comb. n.	H	**N**	New combination
*Phiaris siderana* (Treitschke, 1834)	H	H	
*Selenodes concretana* (Wocke, 1862)	H	H	
*Celypha cespitana* (Hübner, [1817])	H	**P**	Not present in NA
*Argyroploce aquilonana* Karvonen, 1932	H	H	
*Argyroploce externa* (Eversmann, 1844)	H	H	
*Hedya atropunctana* (Zetterstedt, 1840)	H	H	
*Hedya ochroleucana* (Frölich, 1828)	H	**P**	Not present in NA
*Hedya nubiferana* (Haworth, [1811])	I-Eur	I-Eur	
*Hedya salicella* (Linnaeus, 1758)	I-Eur	I-Eur	
*Ancylis comptana* (Frölich, 1828)	H	**P**	Not present in NA
*Ancylis unguicella* (Linnaeus, 1758)	H	H	
*Ancylis uncella* ([Denis and Schiffermüller], 1775)	H	H	
*Eucosmomorpha albersana* (Hübner, [1813])	P	P	
*Enarmonia formosana* (Scopoli, 1763)	I-Eur	I-Eur	
*Rhyacionia buoliana* ([Denis and Schiffermüller], 1775)	I-Eur	I-Eur	
*Spilonota ocellana* ([Denis and Schiffermüller], 1775)	I-Eur	I-Eur	
*Spilonota laricana* (Heinemann, 1863)	I-Eur	I-Eur	
*Eucosma cana* (Haworth, [1811])	P	**H or I?**	
*Eucosma hohenwartiana* ([Denis and Schiffermüller], 1775)	H	H	
*Pelochrista adamantana* (Guenée, 1845)	H	**N**	
*Pelochrista medullana* (Staudinger, 1879)	I-Eur	**P**	Not established in NA
*Epiblema simploniana* Duponchel, 1835)	H	**P**	
*Epiblema arctica* Miller, 1985, stat. rev.	syn. *simploniana*	**N**	Revised status
*Epiblema sticticana* (Fabricius, 1794)	P	**H or I?**	
*Notocelia rosaecolana* (Doubleday, 1850)	I-Eur	I-Eur	
*Notocelia cynosbatella* (Linnaeus, 1758)	I-Eur	I-Eur	
*Gypsonoma parryana* (Curtis, 1835)	H	H	
*Gypsonoma nitidulana* (Lienig and Zeller, 1846)	H	**P**	
*Gypsonoma aceriana* (Duponchel, 1842)	I-Eur	I-Eur	
*Crocidosema plebejana* Zeller, 1847	H	**P**	
*Crocidosema lantana* Busck, 1910	H?	**Neo**	Introduced to Florida
*Rhopobota naevana* (Hübner, [1817])	H	H	
*Epinotia trigonella* (Linnaeus, 1758)	H	H	
*Epinotia solandriana* (Linnaeus, 1758)	H	**H?**	
*Epinotia abbreviana* (Fabricius, 1794)	I-Eur	I-Eur	
*Epinotia nisella* (Clerk, 1759)	H	H	
*Epinotia cinereana* (Haworth, [1811])	H	H	
*Epinotia columbia* (Kearfott, 1904)	H	**N**	
*Epinotia nanana* (Treitschke, 1835)	I-Eur	I-Eur	
*Epinotia medioplagata* (Walsingham, 1895)	H	H	
*Epinotia cruciana* (Linnaeus, 1761)	H	H	
*Epinotia seorsa* Heinrich, 1924	H?	**N**	
*Epinotia salicicolana* Kuznetsov, 1968	I?	**H or I?**	
*Dichrorampha vancouverana* McDunnough, 1935	H	H	
*Dichrorampha sedatana* (Busck, 1906)	H	**H?**	
*Dichrorampha odorata* Brown and Zachariades, 2007	I?	**Neo**	Likely native to Florida
*Dichrorampha petiverella* (Linnaeus, 1758)	I-Eur	I-Eur	
*Dichrorampha acuminatana* (Lienig and Zeller, 1846)	I-Eur	I-Eur	
*Dichrorampha montanana* (Duponchel, 1843)	I-Eur	I-Eur	
*Dichrorampha aeratana* (Pierce and Metcalfe, 1915)	P	**I-Eur**	
*Grapholita molesta* (Busck, 1916)	I-Asia	I-Asia	
*Grapholita delineana* Walker, 1863	I-Asia	I-Asia	
*Grapholita aureolana* (Tengström, 1848)	H	H	
*Cydia coniferana* (Saxesen, 1840)	I-Eur	I-Eur	
*Cydia nigricana* (Fabricius, 1794)	I-Eur	I-Eur	
*Cydia pomonella* (Linnaeus, 1758)	I-Eur	I-Eur	
*Cydia saltitans* (Westwood, 1858), stat. rev.	I	**Neo**	Not present in the U.S.
*Cydia cornucopiae* (Tengström, 1869)	H?	H?	
*Thaumatotibia leucotreta* (Meyrick, 1913)	I	**Afro**	Not established in NA
*Coniostola isabelae* Razowski and Landry, 2008	Neo	Neo	
**Subfamily CHLIDANOTINAE**			
*Auratonota dispersa* Brown, 1990	Neo	Neo	

## Dedication

This work is dedicated to the late William “Bill” E. Miller (1930–2013), a great friend and mentor who spent much of his career researching introduced and Holarctic Tortricidae. Over a span of 60 years he authored 137 publications, most focused on tortricids, and many involving new North American records. He published a series of papers [111,120,121,138] on boreal and Beringian taxa, resolving many issues surrounding taxa that were suspected to be Holarctic. In 1991, as a result of nearly 20 years of experience as the Project Leader at the North Central Forest Experiment Station on the St. Paul campus of the University of Minnesota, he was asked to lead the Lepidoptera section of a U.S. Forest Service project examining pathways and consequences of the introduction of nonindigenous insects. The first author was fortunate enough to inherit all of Bill’s research notes, papers, and book, amounting to several file cabinets full of materials that also included his research on this project. His unpublished report “Immigrant Tortricidae in the continental United States and adjacent area, and in Hawaii” served as the foundation for this paper. Unfortunately, much of his work was outdated by the time we began this study, and Bill did not have the luxury of consulting large databases of DNA data. However, the initial species list for this paper came directly from Bill’s work, along with the criteria used to determine if a species is introduced versus Holarctic, and ultimately the inspiration to complete this project for which Bill had devoted much of his life. We hope that we have accomplished something that would have made Bill proud.
